# Viral Apoptosis Evasion via the MAPK Pathway by Use of a Host Long Noncoding RNA

**DOI:** 10.3389/fcimb.2018.00263

**Published:** 2018-08-03

**Authors:** Samantha Barichievy, Jerolen Naidoo, Mikaël Boullé, Janine Scholefield, Suraj P. Parihar, Anna K. Coussens, Frank Brombacher, Alex Sigal, Musa M. Mhlanga

**Affiliations:** ^1^Gene Expression and Biophysics Group, Synthetic Biology ERA, Council for Scientific and Industrial Research (CSIR), Pretoria, South Africa; ^2^Discovery Sciences, IMED Biotech Unit, AstraZeneca AB R&D, Gothenburg, Sweden; ^3^Division of Chemical Systems and Synthetic Biology, Institute of Infectious Diseases and Molecular Medicine, University of Cape Town, Cape Town, South Africa; ^4^KwaZulu-Natal Research Institute for TB-HIV, Durban, South Africa; ^5^University of KwaZulu-Natal, Durban, South Africa; ^6^Max Planck Institute for Infection Biology Berlin, Germany; ^7^Division of Immunology and South African Medical Research Council Immunology of Infectious Diseases, Faculty of Health Sciences, Institute of Infectious Diseases and Molecular Medicinem University of Cape Town, Cape Town, South Africa; ^8^International Centre for Genetic Engineering and Biotechnology, Cape Town, South Africa; ^9^Division of Medical Microbiology and Institute of Infectious Disease and Molecular Medicine, University of Cape Town, Cape Town, South Africa; ^10^Gene Expression and Biophysics Unit, Instituto de Medicina Molecular, Faculdade de Medicina, Universidade de Lisboa, Lisbon, Portugal

**Keywords:** lincRNA-p21, HuR, hnRNP-K, apoptosis, MAP2K1, ERK2, macrophage, Nutlin3a

## Abstract

An emerging realization of infectious disease is that pathogens can cause a high incidence of genetic instability within the host as a result of infection-induced DNA lesions. These often lead to classical hallmarks of cancer, one of which is the ability to evade apoptosis despite the presence of numerous genetic mutations that should be otherwise lethal. The Human Immunodeficiency Virus type 1 (HIV-1) is one such pathogen as it induces apoptosis in CD4+ T cells but is largely non-cytopathic in macrophages. As a consequence there is long-term dissemination of the pathogen specifically by these infected yet surviving host cells. Apoptosis is triggered by double-strand breaks (DSBs), such as those induced by integrating retroviruses like HIV-1, and is coordinated by the p53-regulated long noncoding RNA lincRNA-p21. As is typical for a long noncoding RNA, lincRNA-p21 mediates its activities in a complex with one of its two protein binding partners, namely HuR and hnRNP-K. In this work, we monitor the cellular response to infection to determine how HIV-1 induces DSBs in macrophages yet evades apoptosis in these cells. We show that the virus does so by securing the pro-survival MAP2K1/ERK2 cascade early upon entry, in a gp120-dependent manner, to orchestrate a complex dysregulation of lincRNA-p21. By sequestering the lincRNA-p21 partner HuR in the nucleus, HIV-1 enables lincRNA-p21 degradation. Simultaneously, the virus permits transcription of pro-survival genes by sequestering lincRNA-p21's other protein partner hnRNP-K in the cytoplasm via the MAP2K1/ERK2 pathway. Of particular note, this MAP2K1/ERK2 pro-survival cascade is switched off during T cell maturation and is thus unavailable for similar viral manipulation in mature CD4+ T cells. We show that the introduction of MAP2K1, ERK2, or HDM2 inhibitors in HIV-infected macrophages results in apoptosis, providing strong evidence that the viral-mediated apoptotic block can be released, specifically by restoring the nuclear interaction of lincRNA-p21 and its apoptosis protein partner hnRNP-K. Together, these results reveal a unique example of pathogenic control over mammalian apoptosis and DNA damage via a host long noncoding RNA, and present MAP2K1/ERK2 inhibitors as a novel therapeutic intervention strategy for HIV-1 infection in macrophages.

## Introduction

Microbial infection, particularly in the case of viral pathogens, often leads to genetic changes in the host chromatin, either via an increase in DNA damage or a decrease in DNA repair activity (Weitzman and Weitzman, 2014). Such genetic instabilities can develop into infection-induced malignancies (de Martel et al., 2012), that are repeatedly linked to classic cancer hallmarks such as enhanced cellular proliferation, and the ability of such genetically damaged cells to evade apoptosis (Hanahan and Weinberg, 2011). For example, the E7 protein from the Human Papillomavirus (HPV) directly binds to the host Retinoblastoma tumor suppressor protein leading to a downstream block in p53-dependent apoptosis (Moody and Laimins, 2010). In a similar fashion, the EBNA3C protein from the Epstein Barr Virus (EBV) directly enhances Aurora kinase B activity which leads to aberrant host cell division and downregulation of apoptosis, all of which occurs despite accumulating DNA damage within these infected B cells (Jha et al., 2013).

In all cases observed thus far, DNA damage that is initiated by pathogenic infection triggers a suite of host cell damage sensor proteins that in turn launch a cascade to recruit repair proteins and form repair response foci (Jackson and Bartek, 2009; Ciccia and Elledge, 2010). This is particularly important in response to double-strand breaks (DSBs), which are the most detrimental form of DNA lesion leading to apoptosis (Jackson and Bartek, 2009). DSBs can result from exposure to ionizing radiation, deoxyribose degeneration via free radicals, inhibition of topoisomerase II, as well as retroviral integration (Jackson and Bartek, 2009). As there is no intact complementary strand to serve as a template for repair, DSBs are poorly tolerated (Khanna and Jackson, 2001), with a single DSB sufficient to kill yeast cells if it inactivates an essential gene, or trigger apoptosis in metazoans (Rich et al., 2000). In mammalian cells, the MRE11/RAD50/NBS1 (MRN) complex senses DSBs leading to activation of the ataxia telangiectasia mutated (ATM) protein kinase and the tumor suppressor protein p53, which plays a central role in the DNA damage response (Meek, 2004; Jackson and Bartek, 2009). Activation of p53 leads to apoptosis, senescence or cell-cycle arrest (Zhou and Elledge, 2000). p53 induces these responses by regulating prosurvival factors such as CDKN1A/p21 as well as several proapoptotic proteins (Riley et al., 2008). Critical to these transcriptional responses is a signaling cascade comprised of serine and threonine kinases that phosphorylate p53 and direct the cell toward apoptosis if the DNA damage remains unrepaired (Siliciano et al., 1997).

Various viruses induce DSBs following infection, and they have evolved a plethora of mechanisms to counteract the ensuing host innate immune response (Weitzman and Weitzman, 2014). This includes viruses that are not directly implicated in causing cancer, although they promote the accumulation of genetic lesions, and target oncogenic hallmark pathways such as apoptosis (Niller et al., 2011). HIV-1 is one such virus, which causes apoptosis in CD4+ T cells driven by the induction of DSBs during the integration step of its life cycle (Doitsh et al., 2010; Cooper et al., 2013). As a retrovirus, HIV-1 cannot exist episomally and must integrate a DNA copy of its proviral genome into the host chromatin following viral-mediated reverse transcription (Craigie and Bushman, 2012). This step is essential for replication and ensures persistence of the virus for the duration of the host cell's survival. Over the normal course of an HIV-1 infection, the progressive loss of CD4+ T cells is so reliable that it is used as a marker of HIV-1 disease progression. Intriguingly, macrophages are also permissive to HIV-1 infection but are largely spared the cytopathic effects of replicating virus, suggesting that there is a selective impairment of the apoptotic response in these particular host cells (Cummins and Badley, 2013). Indeed, several viruses including HIV-1, have been shown to prevent TRAIL-induced apoptosis in macrophages via the control of certain host cytokines (Swingler et al., 2007). However, the roles of cellular integration-associated DSBs and p53, as well as the fundamental molecular mechanism underlying evasion of apoptosis remains unknown.

It is now understood that p53 outsources a critical portion of the host cell's apoptotic response to the long noncoding RNA, lincRNA-p21 (Huarte et al., 2010). In a nuclear complex with its protein binding partner hnRNP-K, lincRNA-p21 orchestrates the apoptotic trigger by specifically repressing the transcription of pro-survival p53 target genes in the nucleus (Huarte et al., 2010). One important target of the lincRNA-p21/hnRNP-K complex is the MAP2K1 gene, whose protein product specifically phosphorylates ERK2 to maintain one of the major cellular survival cascades (Chang and Karin, 2001). Interestingly, activated ERK2 also associates with the HIV-1 pre-integration complex (PIC) thus facilitating successful integration in macrophages (Jacqué et al., 1998; Bukong et al., 2010). In contrast, JNK and Pin1 (and importantly not ERK2) facilitate HIV-1 integration in activated CD4+ T cells (Manganaro et al., 2010). This is possibly because ERK2 expression is specifically and selectively shut down as dual positive (CD4+CD8+) cells go through selection and become CD4+ T cells (Fischer et al., 2005; Chang et al., 2012). As a consequence, ERK2 is unavailable for viral use in CD4+ T cells. Clearly, for HIV-1 to evade apoptosis following integration while simultaneously ensuring survival of the infected host cell, the virus would need to carefully manipulate these pathways. As infecting retroviruses cannot co-ordinate the number of integration events and consequent DSBs per host cell, they must have evolved a separate mechanism to evade the cellular apoptotic response. To achieve this, they would need to either mask the DSBs from the cell or carefully orchestrate any cellular response to the DSB to avoid triggering apoptosis. Furthermore, to induce a DSB yet ensure survival, retroviruses must take control of prosurvival mechanisms and suppress activation of proapoptotic genes.

Both p53-mediated apoptosis, and ERK2-mediated cellular survival, share an intimate connection at the intersection of lincRNA-p21 and MAP2K1. This junction also has a critical connection to HIV via the MAP2K1/ERK2 requirement for viral integration in macrophages. Thus HIV-1 must have evolved a pivotal mechanism to control these host factors as a means to evade apoptosis, with lincRNA-p21 as a central target. In this work, we show how during viral infection of macrophages, HIV-1 is able to cause DSBs without the infected cells apoptosing. We dissect the mechanism of apoptosis evasion and show that HIV-1, in a gp120-dependent manner, triggers sequestration of specific protein binding partners of lincRNA-p21 to different cellular compartments. This ensures lincRNA-p21 transcripts are continually degraded, while simultaneously preventing formation of an active pro-apoptotic nuclear complex. By using a variety of interventions, including anticancer therapeutics, we were able to reverse these effects and demonstrate how control of lincRNA-p21 activity is critical to the apoptosis evasion process of HIV-1 in macrophages. Given that pathogen-induced genetic instability is fairly common, this mechanism is quite likely to be broadly conserved across several host-pathogen interactions, particularly in the context of infection-induced malignancies.

## Materials and methods

### Cell culture

Ghost(3) cells (AIDS Research and Reference Reagent Program, Division of AIDS, NIAID, NIH) were cultured in DMEM (Invitrogen) supplemented with 10% heat-inactivated fetal bovine serum (FBS, Biochrom), 0.2mM GlutaMAX™ (Life Tech), 500 μg/mL G418 (Sigma), 100 μg/mL Hygromycin (Sigma) and 1 μg/mL Puromycin (Sigma). HEK293T cells (AIDS Research and Reference Reagent Program, Division of AIDS, NIAID, NIH) were cultured in DMEM (Invitrogen) supplemented with 10% heat-inactivated FBS (Biochrom). PBMCs from healthy anonymous donors were isolated from buffy coats processed by the Western Province Blood Transfusion Service (University of Cape Town HREC ref 317/2016). PBMCs were separated by density centrifugation over Lymphoprep (Alere). Monocytes were isolated were isolated using Ficoll (GE Healthcare) followed by CD14+ positive selection (Miltenyi microbeads), cultured and differentiated into macrophages for 7 days in RPMI (Invitrogen) supplemented with 10% heat-inactivated Human AB serum (IPLA), sodium pyruvate (Invitrogen), L-Glutamine (Invitrogen) and 20 ng/mL M-CSF (Miltenyi).

### Viral plasmids, virus stocks, infections and drugs

Viral stocks were generated by co-transfecting HEK293T cells with either HIV-1 clones BaL.01 and pSG3Δenv, or NL4-3 and AD8env (AIDS Research and Reference Reagent Program, Division of AIDS, NIAID, NIH) using Fugene6 (Roche). Supernatants were collected 48 h post-transfection, supplemented with FBS to a final concentration of 20% and stored in aliquots at −80°C. HEK293T cell viral stocks were used to infect Ghost(3) cells at an MOI = 1.0 for up to 72 h. For human macrophage infections, HIV-1 strain BaL from National Institute for Biological Standards and Controls was propagated in PBMCs in RPMI-supplemented with 50 mM glutamine, 20% FBS, 100 IU/mL penicillin, 100 μg/mL streptomycin, and 20 IU/mL IL-2 (Sigma) (34). Supernatants were filtered through a 0.22-μM PVDF membrane (Millipore) and purified by ultracentrifugation through a 20% sucrose buffer and resuspended in RPMI medium with 5% human AB serum. M*φ* were infected to a final rate of ~ 4.5% for up to 9 days. Virus input was washed out 24 h post-infection of Ghost(3) cells and 3 days post-infection for M*φ*. Where indicated, cells were treated with 10 μM of either Doxorubicin (Sigma-Aldrich), Nutlin3a (Sigma-Aldrich), MAP2K1 inhibitor (U-0126) or ERK2 inhibitor (FR180204) for up to 48 h.

### Cloning and transfections

Ghost(3) cells were transfected (for 48 h prior to infection) with 25 nM (final concentration) of ON-TARGETplus human HuR/ELAV1 siRNA SMARTpool (5′ GAC AAA AUC UUA CAG GUU U 3′, 5′ GAC AUG UUC UCU CGG UUU G 3′, 5′ ACA AAU AAC UCG CUC AUG C 3′, 5′ GCU CAG AGG UGA UCA AAG A 3′; ThermoScientific) using RNAiMax (Invitrogen). Mouse full-length lincRNA-p21 (3,073 bp) and truncated lincRNA-p21 (1,889 bp) sequences (7) were synthesized (GeneArt, Life Technologies) and sub-cloned via 5′ SacI and 3′ EcoRI into pCi-Neo (Promega). Ghost(3) cells were transfected for 21 h (prior to infection) with either construct using Lipofectamine2000 (Invitrogen).

### Immunofluorescence

For each experiment, cells were infected or treated with drugs on coverslips, fixed for 10 min in fresh 4% paraformaldehyde at room temperature, then washed 3 times in PBS and permeabilized for 10 min in ice-cold methanol at −20°C. Coverslips were washed once in PBS and incubated in blocking buffer (5% goat serum, 0.3% Triton-X100 in PBS) for 60 min at room temperature. Cells were incubated in primary antibody solution (1% BSA, 0.3% Triton X-100 in PBS) overnight at 4°C using the following antibodies rabbit polyclonal anti-phospho-histone H2A.X Ser139 (Cell Signaling), rabbit monoclonal anti-phospho-ATM Ser1981 (Cell Signaling), rabbit polyclonal anti-phospho-p53 Ser46 (Cell Signaling), mouse monoclonal anti-HIV-1 p24 (AIDS Research and Reference Reagent Program, Division of AIDS, NIAID, NIH), mouse monoclonal anti-HuR 3A2 (Santa Cruz Biotechnology), goat polyclonal anti-hnRNP-K P-20 (Santa Cruz Biotechnology), and rabbit polyclonal anti-phospho-MAP2K1/MAP2K2 Ser218/Ser222 (Elabscience Biotechnology). Coverslips were washed 3 times (5 min each on an orbital shaker) with wash buffer (0.05% Tween-20 in PBS), followed by incubation with secondary antibodies conjugated to either Atto-550 or Atto-565 or Atto-647 for 60 min at room temperature. Coverslips were washed 3 times (5 min each on an orbital shaker) with wash buffer (0.05% Tween-20 in PBS). Coverslips were incubated in equilibration buffer (0.4% glucose, 2X SSC) for 5 min and counterstained with 1mg/ml DAPI (4′,6-diamidino-2-phenylindole; Life Technologies). Coverslips were mounted in glox buffer (3.7 mg/ml glucose oxidase, 1U catalase) and imaged. A range of 60 to 600 cells per treatment were imaged as described below.

### Quantitative RT-PCR

For each experiment, cells were infected or treated with drugs over time and total RNA was extracted from cells using TRIzol® (Life Technologies) and Direct-zol^TM^ (Zymo Research), treated with RQ1 RNase-free DNase (Promega) and reverse transcribed using SuperScript III Reverse Transcriptase (Life Technologies). Quantitative RT-PCR was performed using Sso Fast EvaGreen^TM^ supermix (BioRad) on a Bio-Rad CFX96 real-time PCR detection system. PCR primer sets included: p53 (forward 5′ TGTGACTTGCACGTACTCCC 3′, reverse 5′ ACCATCGCTATCTGAGCAGC 3′), CDKN1A/p21 (forward 5′ AGTCAGTTCCTTGTGGAGCC 3′, reverse 5′ GACATGGCGCCTCCTCTG 3′), HIV-1 Gag (5′ ACTCTAAGAGCCGAGCAAGC 3′, reverse 5′ TGTAGCTGCTGGTCCCAATG 3′), lincRNA-p21 (forward 5′ GGGTGGCTCACTCTTCTGGC 3′, reverse 5′ TGGCCTTGCCCGGGCTTGTC 3′), HuR (forward 5′ AGAGCGATCAACACGCTGAA 3′, reverse 5′ TAAACGCAACCCCTCTGG AC 3′), MAP2K1 (forward 5′ ATGGATGGAGGTTCTCTGGA 3′, reverse 5′ TTTCTGGCGACATGTAGGACC 3′) and HPRT (forward 5′ GCAGCCCTGGCGTCGTGATTA 3′, reverse 5′ CGTGGGGTCCTTTTCACCAGCA 3′).

### Apoptosis assay

**Figure 1:**
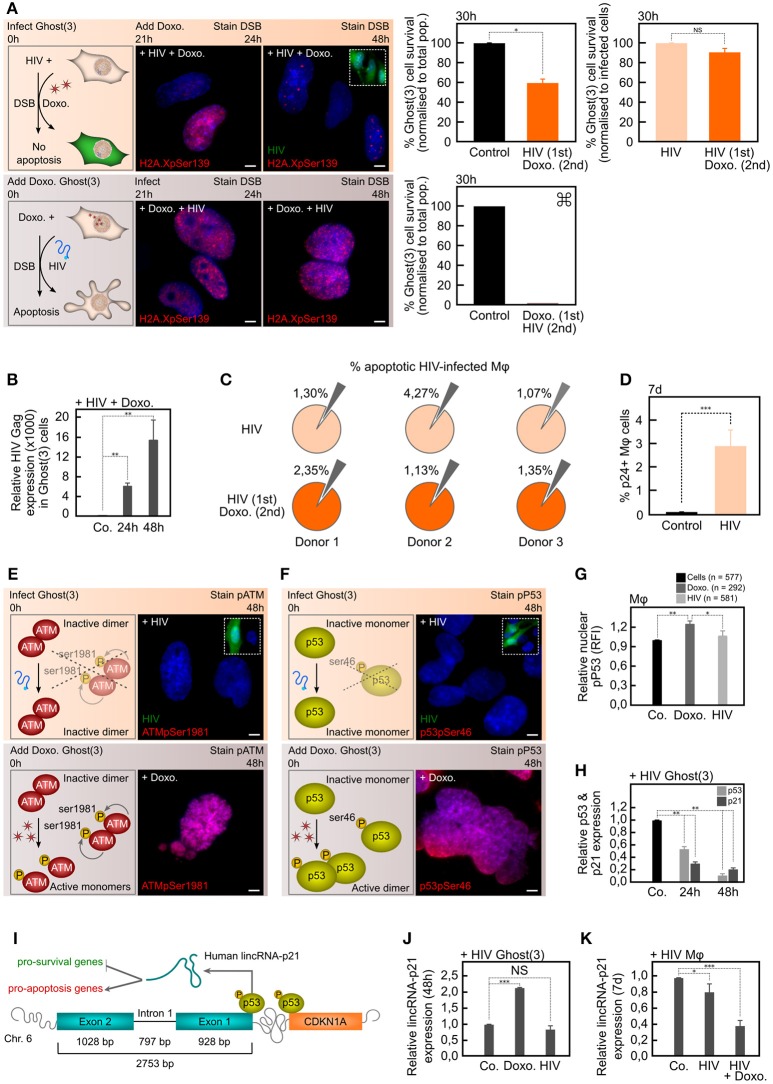
HIV-1 masks DNA damage, protects against additional lethal DNA damage, and prevents lincRNA-p21 upregulation. **(A)** Ghost(3) cells infected 24 h prior to Doxorubicin treatment have numerous DSBs as detected by H2A.XpSer139 immunofluorescence staining (+HIV+Doxo.) but do not undergo apoptosis when normalized to infected cells (HIV 1st Doxo. 2nd; orange bars). Cells infected after exposure to Doxorubicin (+Doxo.+HIV) have extensive H2A.XpSer139 staining and do undergo apoptosis. Too few attached cells present for statistical analysis (<20). **(B)** Ghost(3) cells support HIV-1 replication following additional lethal DNA damage (+HIV+Doxo.) as detected by quantitative real-time RT-PCR analysis of HIV-1 Gag expression relative to the HPRT housekeeping gene and normalized to uninfected cells (mean ± SE of 3 biological replicates in triplicate). **(C)** The percentage of apoptotic HIV-infected M*φ* (small gray wedges, upper row) does not significantly increase in HIV-infected M*φ* exposed to Doxorubicin (small gray wedges, lower row) across 3 separate donors (biological replicates). **(D)** M*φ* were infected with HIV-1 at a physiologically relevant rate as measured at 7 days by p24 flow cytometry analysis (mean ± SE of 3 biological replicates). **(E)** Inactive ATM dimers do not undergo autophosphorylation of serine residue 1981 in response to HIV-induced DSBs as measured by immunofluorescence staining (ATMpSer1981) in Ghost(3) cells. Phosphorylated ATM is detected in Doxorubicin-treated cells. **(F)** Inactive p53 monomers are not phosphorylated at serine residue 46 (specific apoptotic mark) in response to HIV-1 infection of Ghost(3) cells as measured by immunofluorescence staining (p53pSer46). Activated p53 dimers are detected in Doxorubicin-treated cells. **(G)** The relative nuclear fluorescence intensities (RFI) of p53ser46 residues (specific apoptotic mark) is not increased in response to HIV-1 infection of M*φ*, but is increased in Doxorubicin-treated M*φ* (mean ± SE of 3 biological replicates). **(H)** HIV-1 infection significantly decreases p53 and CDKN1A expression over time in Ghost(3) cells as detected by quantitative real-time RT-PCR analysis relative to the HPRT housekeeping gene and normalized to uninfected cells (mean ± SE of 3 biological replicates in triplicate). **(I)** Human lincRNA-p21, which is located upstream of CDKN1A/p21 (orange) on chromosome 6 and comprised of 2 exons (blue) and a single intron, is transcribed by p53 (yellow) in response to DNA damage. LincRNA-p21 mediates cellular apoptosis by down-regulating pro-survival genes (green) and up-regulating pro-apoptosis genes (red). **(J)** HIV-induced DNA damage does not lead to enhanced lincRNA-p21 expression in Ghost(3) cells as detected at 48 h by quantitative real-time RT-PCR analysis relative to the HPRT housekeeping gene and normalized to untreated cells. Doxorubicin treatment does lead to enhanced lincRNA-p21 expression (mean ± SE of 3 biological replicates in triplicate). **(K)** LincRNA-p21 expression is significantly decreased in HIV-infected M*φ* (7 days), with an even greater decrease observed in infected M*φ* exposed to Doxorubicin (HIV+Doxo. at 8 days) as detected by quantitative real-time RT-PCR analysis relative to the HPRT housekeeping gene and normalized to untreated cells (mean ± SE of 3 biological replicates in triplicate). Cells were counterstained with DAPI; scale bars = 10 μM; two-tailed paired Student *T*-test, ***p < 0.001, **p < 0.01, *p < 0.05, NS, not significant; see also Figure S1.

Apoptosis was measured in Ghost(3) cells 30 h post-infection or drug treatment using the NucViewTM 488 Caspase−3 Assay Kit (Biotium) in a 96 well format. Data was analyzed in MATLAB using a probabilistic region method to measure cell attachment and expressed as percentage cell survival. Notably, as the kit was only available with a 488nM dye, cells were analyzed at the 30 h time-point to minimize GFP input from the integrated Tat-driven reporter. An average of 3,000 cells per condition were analyzed with the exception of cells treated with Doxorubicin first followed by HIV-1 infection (Figure 1A) and cells transfected with lincRNA-p21 overexpression constructs followed by Doxorubicin treatment (Figure S2H). These conditions yielded too few attached cells (<20) at 30 h for similar analysis. Apoptosis was measured in M*φ*s 9 days post-infection and/or 2 days after drug treatment using the fixable viability dye Aqua Live/Dead (Thermo Fisher Scientific) in combination with the FLICA 660 caspase 3/7 dye (Thermo Fisher Scientific) after detachment using StemPro® Accutase® (Thermo Fisher Scientific). Cells were then treated with cytofix/cytoperm and perm/wash solutions (BD Biosciences) and stained with FITC-conjugated anti-p24 antibody. A minimum of 10^4^ events were acquired per treatment on a BD LSRForstessa™ flow cytometer.

### RNA pulldown and mass spectrometry

RNA pulldown and mass spectrometry was performed as previously described (Huarte et al., 2010) using Ghost(3) cell lysate and 50pmol of biotinylated probes targeted to lincRNA-p21.

### Imaging and analysis

Cells were imaged on one of two microscopes: (1) a customized Nikon Ti Eclipse widefield fluorescent microscope using a 100 × 1.49 N.A. Nikon Apochromat TIRF oil immersion objective, with mercury lamp illumination through the appropriate Semrock razor sharp filter sets at low camera gain in each of the fluorescent channels using an Andor iXion 897 EMCCD camera cooled to −80°C, and controlled using μmanager open source microscope management software (NIH and UCSF, USA). A 30 ms exposure time was used for DAPI. Exposure times ranged from 200 to 500 ms for other dyes. Each field of view was captured as a series of images acquired on multiple focal planes through the samples, across a range of 2–10 mm in the axial plane. A 0.2 mm piezo step-size was used for z-stacks. (2) An Andor Technology (Belfast, Northern Ireland) integrated Yokogawa CSU-W1 spinning disk confocal system and a Metamorph controlled Nikon TiE motorized microscope with a 60 ×, 1.4 NA phase oil immersion objective. Excitation sources were 405, 488, and 561 laser lines and emissions were detected through Semrock Brightline 465, 525, and 607 nm filters. Images were captured using an 888 EMCCD camera (Andor) and iQ2 software. Eight fields of view were captured per well (two wells per condition, from 3 donors), eight Z-sections per FOV with one phase and three fluorescent images. Signal intensities of all images were measured using Fiji (Schindelin et al., 2012). The contrast of images shown was adjusted to fit a 16 bit gray-scale.

## Results

### HIV masks DNA damage

Given that HIV-1 causes apoptosis in CD4+ T cells following integration (Doitsh et al., 2010; Cooper et al., 2013), yet is able to extend survival and prevent elimination of infected macrophages (Swingler et al., 2007), it is likely that the virus can control the cellular response to apoptosis. This viral-induced cellular ability to survive despite DNA damage suggests that such cells can be exposed to, yet withstand, significant genotoxic stress. In support of this, chronically infected U937 monocyte-like cells are more resistant to genotoxic DNA-damaging agents (Tanaka et al., 1999). Thus we reasoned that in HIV-infected cells, only a portion of the cellular DSB surveillance mechanism may be intact. To ascertain this, we added a lethal dose of the DNA-damaging agent Doxorubicin to HIV-infected Ghost(3) reporter cells or primary monocyte-derived macrophages (M*φ*) and monitored the development of DSBs and apoptosis.

The Ghost(3) reporter cells allowed us to monitor early infection events via the integrated reporter which consists of an HIV-1 LTR promoter fused to GFP (Figure S1A). Following integration, host-mediated transcription of the proviral genome leads to the production of Tat, which in turn promotes processive transcription from the LTR and the production of GFP by 24 h and nascent virions at 48 h (Figure S1B). Active viral replication following integration and the related DSBs were visualized in Ghost(3) cells by immunofluorescent staining of H2A.X (Figure 1A, Figures S1C–E). This histone variant is rapidly phosphorylated at serine 139 (Ser139) within seconds of such DNA damage and persists until the DSB is repaired (Keogh et al., 2006). By concurrently assessing the production of activated caspase 3 as a marker of apoptosis induction (shown as percentage cell survival), we observed that cells which received lethal doses of Doxorubicin prior to HIV-1 infection (Doxo. 1st HIV 2nd), underwent apoptosis (~0% survival, Figure 1A, lower panel). This was expected given that Doxorubicin is genotoxic in isolation and adding virus subsequent to such genomic stress was not expected to rescue these cells.

However, cells infected 24 h before being exposed to the same duration and concentration of Doxorubicin treatment (HIV 1st Doxo. 2nd) were protected from apoptosis (60% survival, Figure 1A, upper panel). Notably, survival in HIV-infected cells exposed to Doxorubicin was the same as HIV-1 infection alone (Figure 1A, upper panel), although viral replication was lower in the former conditions (Figure 1B, 16,000 Gag units vs. Figure S1B, 48,000 Gag units). Remarkably, the same apoptosis-resistance phenotype was observed in Doxorubicin-treated M*φ* across 3 separate donors (Figure 1C) each infected at physiologically relevant levels (Figure 1D). Overall these data independently supported the previous observations in infected U937 cells (Tanaka et al., 1999) showing that infected cells are protected from additional genotoxic stress. As we could observe DSBs following infection plus the resulting apoptosis induction yet survival of such cells, we could also conclude that the virus is able to decouple the DNA damage surveillance mechanism from the apoptotic response.

The cellular response to DSBs is exquisitely sensitive and DNA damage is recognized by ATM, which is activated by auto-phosphorylation of Ser1981 (Lee and Paull, 2005). A key downstream target of activated ATM is p53, which is phosphorylated at multiple residues (Riley et al., 2008) including Ser46. Phosphorylation at Ser46 specifically directs cellular responses toward apoptosis (Smeenk et al., 2011). As DSBs are known to trigger ATM and p53 activation, we assessed their specific phosphorylation states in response to viral and chemically induced DNA damage. Doxorubicin treatment resulted in the robust phosphorylation of both ATMpSer1981 (Figure 1E) and p53pSer46 (Figure 1F) within 8 h in the nuclei of Ghost(3). In contrast, HIV-1 infection did not lead to phosphoryation of either of these residues over 48 h. Similarly, the same major apoptotic mark on nuclear-localized p53 was strikingly enhanced in Doxorubicin-treated M*φ* as opposed to those exposed to HIV-1 (Figure 1G, Figure S1F). Furthermore, quantitative real-time RT-PCR analysis revealed that expression of p53, as well as one of its major downstream cell cycle factors CDKN1A/p21 (Wu et al., 2010), was significantly decreased during the same time course in Ghost(3) cells in the presence of virus (Figure 1H). Taken together, these observations provide further evidence that HIV-1 is able to carefully orchestrate the cellular response to DSBs very early on thereby avoiding apoptosis, and suggests that manipulation of p53 is central to this process.

### HIV negatively affects a host long noncoding RNA regulating apoptosis

A critical portion of the p53-mediated apoptotic transcriptional response was recently shown to be controlled by the long intergenic noncoding RNA lincRNA-p21 (Huarte et al., 2010). In response to DNA damage, p53 transcriptionally activates lincRNA-p21 which is located on chromosome 6 upstream of CDKN1A/p21 in human cells (Figure 1I; Huarte et al., 2010; Yoon et al., 2012). Since HIV-1 had neutralized the apoptotic response in both Ghost(3) cells and primary M*φ*, we examined the status of lincRNA-p21 in HIV-infected and control cells. As previously noted (Huarte et al., 2010; Yoon et al., 2012), lincRNA-p21 was enhanced in Doxorubicin-treated cells, and intriguingly HIV-infected Ghost(3) cells showed no increase in lincRNA-p21 expression (Figure 1J). This latter observation was also true of viral-infected M*φ*, and even more notable was that in the presence of virus plus additional lethal DNA damage (Doxorubicin treatment), lincRNA-p21 levels were further decreased (Figure 1K). Given the central role of this host long noncoding RNA in regulating apoptosis, these data strongly suggested that HIV-1 may prevent this cellular response by direct or indirect regulation of lincRNA-p21.

**Figure 2:**
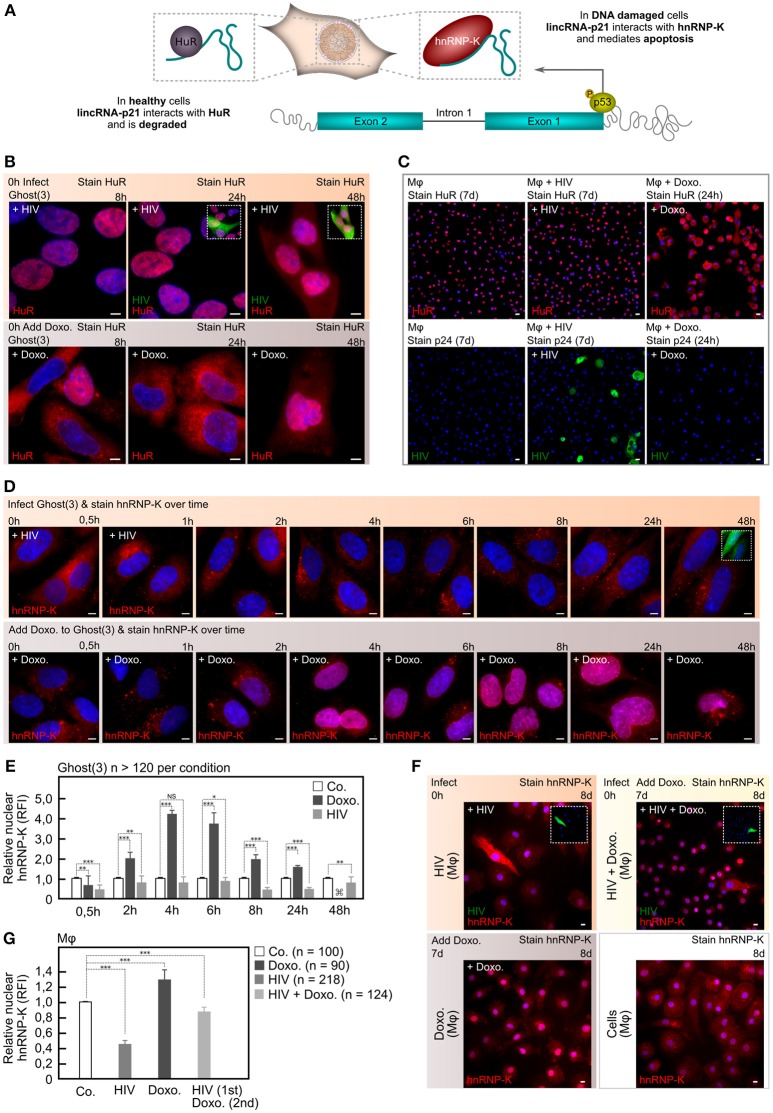
HIV-1 manipulates lincRNA-p21's protein binding partners. **(A)** In healthy cells, lincRNA-p21 associates with HuR in the nucleus and is degraded. In response to p53-mediated transcription, lincRNA-p21 associates with hnRNP-K in the nucleus of DNA-damaged cells and regulates apoptosis by localizing hnRNP-K to the promoters of p53-repressed genes. **(B)** Immunofluorescence staining reveals cytoplasmic HuR within 8 h of Doxorubicin-treated Ghost(3) cells while HIV-infected cells show nuclear HuR up until 48 h. **(C)** Immunofluorescence staining reveals nuclear HuR in untreated and HIV-infected M*φ* (counterstained with p24 in lower panel), but clear cytoplasmic HuR in Doxorubicin-treated M*φ*. **(D)** Immunofluorescence staining reveals nuclear hnRNP-K within 2 h of Doxorubicin treatment in Ghost(3) cells, while HIV-infected cells show cytoplasmic hnRNP-K throughout all 8 time points spanning the same 48 h time course. **(E)** Quantification of nuclear localized hnRNP-K in HIV-infected Ghost(3) cells over time (mean relative fluorescence intensity (RFI) ± SE of 3 biological replicates). Too few attached cells were present for statistical analysis (<20). **(F)** Immunofluorescence staining reveals nuclear hnRNP-K within 24 h of Doxorubicin treatment of M*φ* (Doxo.), while HIV-infected cells (+HIV) and those subsequently exposed to Doxorubicin (+HIV+Doxo.) show cytoplasmic hnRNP-K. **(G)** Quantification of nuclear localized hnRNP-K in M*φ* infected with HIV-1 or infected and exposed to Doxorubicin (HIV 1st, Doxo. 2nd); mean relative fluorescence intensity (RFI) ± SE of 3 biological replicates). Cells were counterstained with DAPI; scale bars = 10 μM; two-tailed paired Student *T*-test (**G** used two-tailed unpaired Student *T*-test), ***p < 0.001, **p < 0.01, *p < 0.05, NS, not significant; see also Figure S2.

Under normal cellular conditions, lincRNA-p21 is rendered unstable through the action of HuR/ELAV1 in the nucleus (Figure 2A; Yoon et al., 2012). More specifically, this occurs via HuR/ELAV1 mediated degradation of lincRNA-p21 through the recruitment of the Ago2 protein and let-7a mirRNA. Thus in normal healthy cells, lincRNA-p21 levels remain low via its interactions with HuR/ELAV1, and the pro-apoptotic effects of the long noncoding RNA are negated. Based on our observation that HIV-1 was able to ensure survival in host cells despite the presence of lethal DNA damage, we hypothesized that HIV-1 may co-opt the HuR/ELAV1 degradation mechanism to maintain lincRNA-p21 instability and thus evade apoptosis. Our hypothesis was initially bolstered by the observation of a significant difference in the cellular location of HuR in M*φ* and Ghost(3) cells infected with HIV-1 as compared to chemically induced DSBs (Figures 2B,C, Figure S2A). Indeed during HIV-1 integration and concomitant induction of DSBs (Figure S1C), HuR remained in the nucleus over the course of infection, presumably maintaining its destabilizing effect on lincRNA-p21. Contrastingly, in Doxorubicin-treated cells, HuR moved from the nucleus into the cytoplasm within 8 h of treatment, thus releasing the destabilizing effect on lincRNA-p21. This latter observation was also consistent with the lincRNA-p21 expression levels we had previously detected with Doxorubicin treatment being the only condition that showed elevated lincRNA-p21 (Figures 1J,K).

Since HIV-infected cells failed to apoptose despite lethal levels of DNA damaging agents, and lincRNA-p21 levels were lowered by nuclear resident HuR, we reasoned that silencing HuR should elevate lincRNA-p21 and subsequently trigger apoptosis (Yoon et al., 2012). We delivered siRNAs targeted to HuR in HIV-infected Ghost(3) cells and observed a rise in lincRNA-p21 as detected by qRT-PCR (Figures S2B,C). However, siHuR-treated cells continued to support HIV-1 replication to the same extent as untreated cells (Figures S2D,E). These results suggested that although the virus clearly manipulates the cellular location of HuR in order to control lincRNA-p21 expression, this alone could not account for the block in apoptosis observed in infected cells. As an alternative strategy, we exogenously boosted lincRNA-p21 expression in the presence of HIV-1. Notably, overexpressed full-length lincRNA-p21 was decreased in the presence of HIV-1 in Ghost(3) cells and did not lead to apoptosis, whereas similarly transfected cells treated with Doxorubicin underwent apoptosis (Figures S2F–H). Collectively these data showed that while HIV-1 uses the location of HuR to maintain low lincRNA-p21 expression levels, there must be additional molecular mechanisms by which the virus is able to evade apoptosis.

### HIV alters the protein binding partners of a pro-apoptotic host lncRNA

The interplay between p53, lincRNA-p21 and hnRNP-K is now understood to be cause repression of a specific set of genes that are part of the p53 DNA damage response (Huarte et al., 2010). Key to this repression is the formation of a lincRNA-p21/hnRNP-K complex specifically in the nucleus of cells (Huarte et al., 2010). Since nuclear localization of hnRNP-K is required for its repressive function, and as HIV-1 is able to avoid apoptosis, we hypothesized that in addition to reducing nuclear levels of lincRNA-p21, the virus was altering hnRNP-K's cellular location within infected cells. We monitored hnRNP-K at several time points following DNA damage induced either by Doxorubicin treatment or HIV-1 infection using immunofluorescence staining (Figures 2D–G). HnRNP-K was detected in the nuclei of Doxorubicin-treated Ghost(3) cells between 2 and 4 h post-treatment and thereafter continued to accumulate in the same location until apoptosis occurred between 24 and 48 h. In contrast, hnRNP-K remained largely cytoplasmic in HIV-infected cells throughout the duration of the time course with no apoptosis occurring despite clear and active HIV-1 infection (Figures 2D,E).

HIV-infected M*φ* showed an identical phenotype with cytoplasmic localization of hnRNP-K in viral infected cells in contrast to nuclear hnRNP-K in Doxorubicin treated cells (Figures 2F,G). Furthermore, infected M*φ* subsequently exposed to lethal Doxorubicin treatment showed significantly less nuclear hnRNP-K as compared to untreated cells or those treated with Doxorubicin alone (Figures 2F,G). These observations parsimoniously explained the inability to trigger transcription of pro-apoptotic genes, since hnRNP-K was unable to associate with lincRNA-p21 in the nucleus. It also explained why the addition of exogenous lincRNA-p21 is unable to induce apoptosis in HIV-infected cells. In support of this, in addition to HuR and hnRNP-K, HIV-1 infection altered the binding of multiple additional protein partners of lincRNA-p21 (assessed by IP-MS, Figures S2I,J), further underpinning our hypothesis that HIV-1 alters the lincRNA-p21/hnRNP-K interaction to avert apoptosis.

### HIV controls a pro-apoptotic host lncRNA via MAPK pathway members

**Figure 3:**
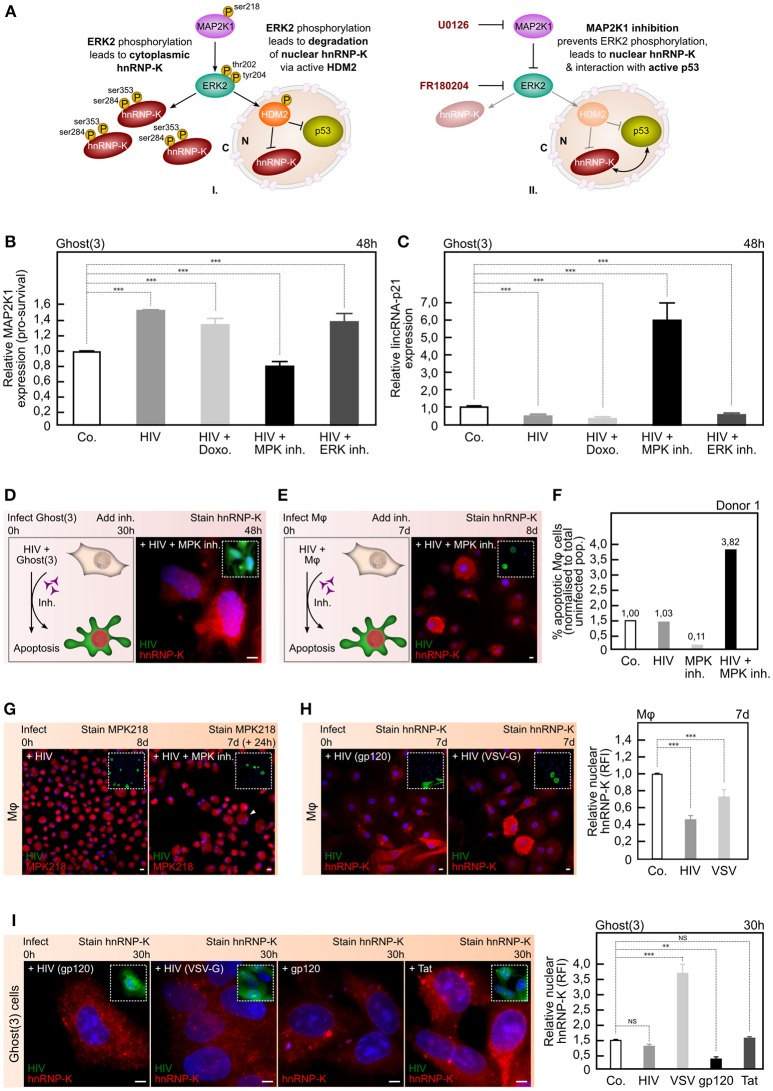
HIV-1 requires MAP2K1/ERK2 and gp120 to ensure hnRNP-K's cytoplasmic localization. **(A)** Activated MAP2K1 specifically phosphorylates ERK2 leading to cytoplasmic accumulation of phosphorylated hnRNP-K (I). Simultaneously, ERK2 activation of HDM2 ensures ubiquitin-mediated degradation of nuclear hnRNP-K and p53 (I). Inhibition of MAP2K1 (or ERK2) prevents ERK2-mediated accumulation of hnRNP-K, and releases the negative regulation of HDM2 on nuclear hnRNP-K and p53 (II). **(B)** HIV-1 enhances MAP2K1 expression in Ghost(3) cells, in the presence of Doxorubicin (HIV+Doxo.) or an ERK2 inhibitor (HIV+ERK inh.) but not in the presence of a MAP2K1 inhibitor (MPK inh.) as detected by quantitative real-time RT-PCR analysis relative to the HPRT housekeeping gene and normalized to untreated cells (mean ± SE of 3 biological replicates in triplicate). **(C)** HIV-1 requires enhanced MAP2K1 expression to reduce lincRNA-p21 levels in Ghost(3) cells as only in the presence of a MAP2K1 inhibitor (HIV+MPK inh.) is lincRNA-p21 expression enhanced, as detected by quantitative real-time RT-PCR analysis relative to the HPRT housekeeping gene and normalized to untreated cells (mean ± SE of 3 biological replicates in triplicate). **(D)** Inhibition of MAP2K1 allows for nuclear localization of hnRNP-K and apoptosis in HIV-infected Ghost(3) cells as measured by immunofluorescence staining. **(E)** Inhibition of MAP2K1 allows for nuclear localization of hnRNP-K and apoptosis in HIV-infected M*φ* as measured by immunofluorescence staining. **(F)** The percentage of apoptotic M*φ* (normalized to total uninfected population) increases in HIV-infected M*φ* exposed to a MAP2K1 inhibitor (HIV+MPK inh.). Data shown for 1 of 3 donors. **(G)** Immunofluorescence staining of the Ser218/222 activation mark on MAP2K1 in HIV-infected M*φ* shows punctate cytoplasmic localization of the protein, only in the presence of the inhibitor (white arrowhead). **(H)** HIV-1 clone NL4-3 packaged with gp120 Env (+HIV gp120) excludes hnRNP-K from the nucleus of infected M*φ* as shown by immunofluorescence staining, while HIV-1 packaged with VSV-G (+HIV VSV-G) fails to do so. Quantification of nuclear localized hnRNP-K in infected M*φ* shown in right panel (mean relative fluorescence intensity (RFI) ± SE of 3 biological replicates). (I) HIV-1 clone BaL.01 packaged with gp120 Env (+HIV gp120) excludes hnRNP-K from the nucleus of infected Ghost(3) cells as shown by immunofluorescence staining, while HIV-1 packaged with VSV-G (+HIV VSV-G) fails to do so. Cells transfected with a plasmid expressing only HIV-1 gp120 (+gp120) show cytoplasmic hnRNP-K, while control cells transfected with a plasmid expressing only HIV-1 Tat (+Tat) show cytoplasmic and nuclear hnRNP-K. Quantification of nuclear localized hnRNP-K in treated Ghost(3) cells shown in right panel [mean relative fluorescence intensity (RFI) ± SE of 3 biological replicates]. Cells were counterstained with DAPI; scale bars = 10 μM; two-tailed paired Student *T*-test, ***p < 0.001, **p < 0.01, NS, not significant; see also Figure S3.

In healthy cells MAP2K1 is the primary kinase that activates ERK2 as part of a canonical survival cascade (Habelhah et al., 2001; Cagnol and Chambard, 2010). Following activation, ERK2 specifically phosphorylates hnRNP-K at serines 284 and 353, leading specifically to the cytoplasmic accumulation of hnRNP-K (Habelhah et al., 2001). Inhibition of the MAP2K1/ERK2 pathway prevents this series of phosphorylation events on hnRNP-K and thus affects hnRNP-K cellular location (Figure 3A, Habelhah et al., 2001). As MAP2K1 is a key target of the lincRNA-p21/hnRNP-K complex and MAP2K1 transcription is thus negatively regulated as part of this pro-apoptosis pathway (Huarte et al., 2010), we sought to determine if HIV-1 secures cellular survival via MAP2K1/ERK2.

Initially we examined MAP2K1 transcription and found its expression to be significantly up-regulated in the presence of HIV-1 as compared to control cells (Figure 3B, Figure S3A). Intriguingly, this was also observed in HIV-infected cells that were subsequently exposed to a lethal dose of Doxorubicin suggesting that the MAP2K1/ERK2 survival cascade was still actively switched on in these cells, at least at the level of MAP2K1 expression (Figure 3B, Figure S3A). When we added a MAP2K1-specific inhibitor (thus preventing specific downstream ERK2 activation) to HIV-infected cells, MAP2K1 expression was significantly reduced as expected (Figure 3B, Figure S3A). In addition, infected cells treated with an ERK2-specific inhibitor showed enhanced MAP2K1 expression relative to control cells (Figure 3B, Figure S3A). Together, these observations revealed that HIV-1 infection specifically increases MAP2K1 expression, and strongly suggested that HIV-1 can counteract lincRNA-p21 mediated suppression of MAP2K1.

To ascertain this, we next examined lincRNA-p21 expression under similar conditions, and observed that HIV-infected cells treated with a MAP2K1 inhibitor (hence no activated ERK2) showed significantly increased lincRNA-p21 levels (Figure 3C, Figure S3B). In contrast, infected cells treated with an ERK2 inhibitor did not show increased lincRNA-p21 (Figure 3C, Figure S3B). Notably, in HIV-infected cells subsequently treated with Doxorubicin, lincRNA-p21 levels were also significantly reduced (Figure 3C, Figure S3B). These results were in line with our observed changes in MAP2K1 expression and clearly demonstrated that HIV-1 is able to suppress lincRNA-p21 expression by ensuring high levels of MAP2K1 expression.

Having established a lincRNA-p21/MAP2K1 connection in the presence of virus, we then sought to determine how HIV-mediated manipulation of MAP2K1 expression affected hnRNP-K location and cellular survival. HIV-infected Ghost(3) cells (Figure 3D, Figures S3C,D) and M*φ* (Figure 3E, Figures S3E,F) exposed to a MAP2K1 inhibitor revealed strong nuclear localization of hnRNP-K as measured by immunofluorescence, with infected M*φ* undergoing apoptosis in the presence of the inhibitor (Figure 3F, Figure S3G). This observation was striking and suggested that MAP2K1 is a key host factor required by the virus to subvert lincRNA-p21-mediated apoptosis. These results could not rule out a possible role for ERK2, which is known to interact with the pre-integration complex (Jacqué et al., 1998; Bukong et al., 2010) as well as Tat soon after integration (Wolf et al., 2008). However, as apoptosis studies involving ERK2 are complicated by an unusually prolonged presence of activated (pro-survival) ERK2, specifically in the presence of the MAP2K1 inhibitor U0126 and Doxorubicin (Cagnol and Chambard, 2010), we did not monitor this host factor further. Instead, immunofluorescence staining of the Ser218/222 activation mark on MAP2K1 in HIV-infected M*φ* showed distinct punctate cytoplasmic localization of the protein, only in the presence of the inhibitor (Figure 3G, Figure S3H). This was in line with prior art showing that aberrant sub-cellular localization of MAP2K1/ERK pathway members is directly associated with cell death (Liu et al., 2008; Cagnol and Chambard, 2010). Taken together, these findings strongly supported our hypothesis that HIV-1 requires MAP2K1/ERK2 not only for integration but also to ensure cytoplasmic accumulation and sequestration of hnRNP-K, and therefore cellular survival. Given that MAP2K1 transcription is negatively regulated via the lincRNA-p21/hnRNP-K complex, by securing positive MAP2K1 signaling in concert with integration, HIV-1 is able to prevent nuclear entry of hnRNP-K, and thus prevent apoptosis.

Having identified the specific host protein manipulated by HIV-1 to ensure cytoplasmic hnRNP-K, we next sought to determine which viral protein could be mediating this effect. Our previous result led us to believe that HIV-1 is likely manipulating a host factor very early on after infection given the role of ERK2 in integration (Jacqué et al., 1998; Bukong et al., 2010) and cellular survival immediately downstream of MAP2K1. We thus began with simply switching the native gp120 envelope protein for vesicular stomatitis virus G (VSV-G) envelope protein. Interestingly, virus pseudotyped with VSV-G was unable to exclude hnRNP-K from the nuclei of both M*φ* (Figure 3H) and Ghost(3) cells (Figure 3I) suggesting that gp120 itself was involved. Supporting this observation, Ghost(3) cells transfected with gp120 envelope alone showed cytoplasmic hnRNP-K (Figure 3I). Intriguingly, gp120 also affected HuR, such that VSV-G pseudotyped HIV-1 did not exclude HuR from the cytoplasm, and gp120 alone led to nuclear HuR (Figure S3I). Furthermore, VSV-G pseudotyped HIV-infected M*φ* showed greater levels of apoptosis in the presence of a MAP2K1 inhibitor as compared to HIV-gp120 infected counterparts (Figure S3G). Together, these data strongly suggested that viral control of hnRNP-K's cellular location requires gp120, and further demonstrated that HIV-1 gains control of hnRNP-K and HuR very early on during infection.

### Restoring lncRNA/protein partner interactions in infected cells induces apoptosis

To confirm our earlier findings, and conclusively demonstrate that cytoplasmic accumulation of hnRNP-K plays a central role in the mechanism HIV-1 uses to evade apoptosis, we interrogated the pathway by using drugs that regulate hnRNP-K activity in response to DNA damage and p53-mediated apoptosis. We designed an experiment by which we could restore hnRNP-K to the nucleus during HIV-1 infection and concurrently boost lincRNA-p21 levels. We reasoned that overcoming this block would help to re-establish hnRNP-K's interaction with lincRNA-p21 and trigger cellular apoptosis in infected cells.

**Figure 4:**
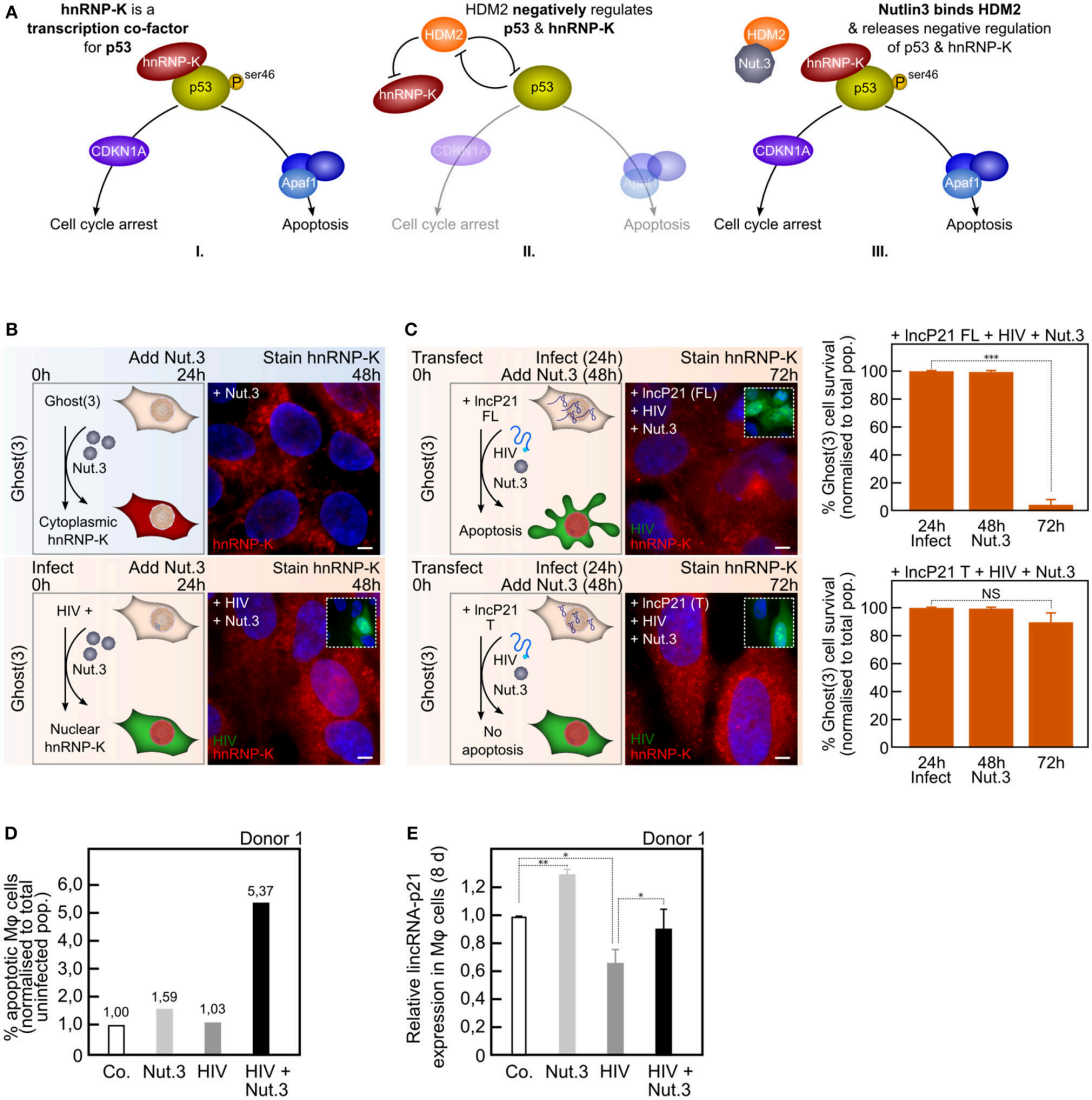
Nutlin3a confirms pivotal role of hnRNP-K in apoptosis evasion by HIV-1. **(A)** In response to DNA damage, both p53 and hnRNP-K are activated with cells undergoing apoptosis if p53ser46 is phosphorylated (I). In healthy cells, both p53 and hnRNP-K are negatively regulated by HDM2 (II). Nutlin3a (Nut.3) binds HDM2 thereby releasing its negative regulation of p53 and hnRNP-K (III). **(B)** Exposure of Ghost(3) cells to Nutlin3a 24 h post-infection leads to nuclear localization of hnRNP-K at 48 h in infected (GFP-positive) cells only (+HIV+Nut.3a). Control cells show cytoplasmic hnRNP-K as expected in the absence of DNA damage. **(C)** Exogenous full-length lincRNA-p21 expression and Nutlin3a treatment lead to nuclear hnRNP-K and apoptosis in HIV-infected Ghost(3) cells (+lncP21-FL+HIV+Nut.3). Similarly treated cells transfected with truncated exogenous lincRNA-p21 show nuclear hnRNP-K but do not undergo apoptosis (+lncP21-T+HIV+Nut.3). **(D)** The percentage of apoptotic M*φ* (normalized to total uninfected population) significantly increases only in HIV-infected M*φ* exposed to Nutlin3a (HIV+Nut.3). Data shown for 1 of 3 donors. **(E)** LincRNA-p21 expression is significantly increased in HIV-infected M*φ* exposed to Nutlin3a (HIV+Nut.3a) when compared to HIV-infection alone (+HIV) as detected by quantitative real-time RT-PCR analysis relative to the HPRT housekeeping gene and normalized to untreated cells (mean ± SE of 1 biological replicate in triplicate). Cells were counterstained with DAPI; scale bars = 10 μM; two-tailed paired Student *T*-test, **p < 0.01, *p < 0.05, see also Figure S4.

Important insight came from prior art that describes hnRNP-K in connection to DNA damage. Following genotoxic stress various signaling cascades are activated, one of which culminates in hnRNP-K activation chiefly through inhibition of its degradation by the E3 ligase HDM2 (Moumen et al., 2005). A separate signaling cascade leads to activation of p53, also chiefly through inhibition of its degradation by HDM2 Enge et al., 2009). Both p53 and hnRNP-K are thus activated in response to DNA damage and both are negatively regulated through the action of HDM2. Nutlin3a is a small molecule inhibitor that activates p53 by binding to HDM2 (Vassilev et al., 2004). As Nutlin3a had previously been linked to activation of hnRNP-K (Moumen et al., 2005), we hypothesized that treatment with this inhibitor may allow hnRNP-K to move into the nucleus of HIV-infected cells (Figure 4A). To begin with, we treated control Ghost(3) cells with Nutlin3a in the absence of any DNA damage and observed no nuclear hnRNP-K (Figure 4B, upper panel). This suggested that even if the negative HDM-mediated regulation of hnRNP-K is released, DNA damage is still required to trigger nuclear localization of hnRNP-K (Figure 4B, upper panel). In contrast, Nutlin3a treatment following 24 h of infection resulted in nuclear localization of hnRNP-K in HIV-infected Ghost(3) cells only (Figure 4B, lower panel). This result demonstrates that the addition of Nutlin3a after infection is sufficient to prevent the virus from excluding hnRNP-K from the nucleus of Ghost(3) cells.

Since HIV-1 infection lowers lincRNA-p21 levels by HuR-mediated degradation (Figures 2B,C), overcoming the nuclear exclusion of hnRNP-K seemed insufficient to induce apoptosis (Figure 4B). We thus sought to use Nutlin3 to enable nuclear localization of hnRNP-K while concomitantly up-regulating lincRNA-p21 levels. This was technically feasible in Ghost(3) cells and importantly, we initially established that overexpressed lincRNA-p21 by itself did not lead to nuclear hnRNP-K and apoptosis in HIV-infected Ghost(3) cells (Figures S4A,B). Following this, we transfected Ghost(3) cells for 24 h with lincRNA-p21, infected for the next 24 h, and followed with Nutlin3a treatment. Notably, only in the presence of full-length lincRNA-p21 did HIV-infected Ghost(3) cells treated with Nutlin3a demonstrate nuclear hnRNP-K and undergo apoptosis (Figure 4C, upper panel). In contrast, cells that received a truncated version of lincRNA-p21, which is unable to bind hnRNP-K (Huarte et al., 2010), showed nuclear hnRNP-K but did not undergo apoptosis (Figure 4C, lower panel).

Furthermore, we verified that the combination of overexpressed lincRNA-p21 and Nutlin3a in the absence of HIV-1 did not lead to nuclear localization of hnRNP-K and apoptosis in Ghost(3) cells (Figures S4B,C). Crucially, while exogenous overexpression of full-length or truncated lincRNA-p21 was not possible in M*φ*, the addition of Nutlin3a in the presence of virus induced apoptosis (Figure 4D, Figure S4D). In addition, this effect was particularly evident in M*φ* infected with VSV-G pseudotyped HIV-1 (Figure S4D), in line with our previous data showing that gp120 plays a crucial role in the virus' ability to evade hnRNP-K mediated apoptosis. Finally, lincRNA-p21 levels were concomitantly upregulated in HIV-infected M*φ* treated with Nutlin3a (Figure 4E, Figure S4E).

Overall, these results indicate that restoring hnRNP-K to the nucleus in the presence of elevated full-length lincRNA-p21, leads to apoptosis in HIV-infected cells. In addition, the observation that truncated lincRNA-p21 did not similarly result in apoptosis strongly supports the agency of lincRNA-p21 in this process, confirming the requirement of a fully functional binding complex comprised of lincRNA-p21 and hnRNP-K (Figure S4F; Huarte et al., 2010). Furthermore, the enhanced apoptosis levels observed in the presence of VSV-G pseudotyped virus firmly support the role of gp120 in this mechanism. In sum, these results highlight the pivotal role that a host long noncoding RNA plays in HIV-1 apoptosis evasion mechanisms.

## Discussion

**Figure 5:**
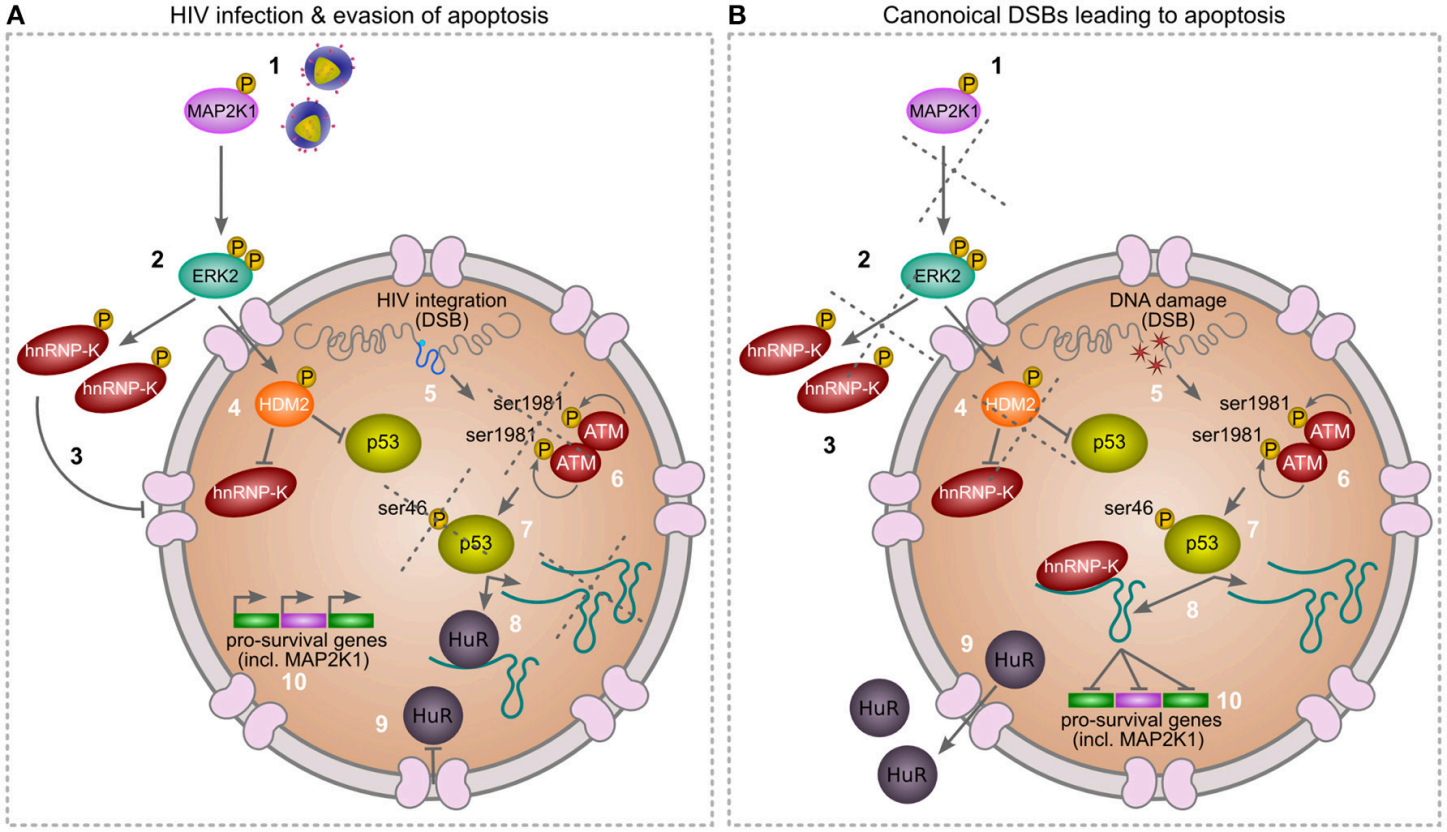
Hypothetical model of HIV-mediated manipulation of lincRNA-p21 to evade cellular apoptosis (comparing **A,B**). During canonical DNA damage, the MAP2K1/ERK2 pathway is inactivated. However, during infection, HIV-1 ensures activated MAP2K1 (1) continues to phosphorylate ERK2 (2) thereby leading to cytoplasmic accumulation of hnRNP-K (3) as well as phosphorylation of HDM2 (4). The latter action of ERK2 ensures ubiquitin-mediated degradation of nuclear hnRNP-K as well as p53. When compared to canonical DNA damage, both HIV-1 integration and Doxorubicin induce DSBs (5). However, in HIV-infected cells this does not lead to autophosphorylation and activation of ATM at serine1981 (6) or subsequent phosphorylation of p53 at serine46 (7). The p53pSer46 apoptosis mark leads to increased lincRNA-p21 transcription and association with hnRNP-K (8), as well as translocation of HuR to the cytoplasm (9) of canonically DNA damaged cells only. Nuclear lincRNA-p21/hnRNP-K complexes lead to suppression of pro-survival genes in canonically DNA damaged cells only (10). As HIV-1 is able to alter the location of hnRNP-K (3) and HuR (9), lincRNA-p21 expression is low and pro-survival genes, including MAP2K1, are not repressed in infected cells (10), thus the virus is able to evade apoptosis.

Our findings elucidate the deliberate and focused attack HIV-1 aims at lincRNA-p21 to mask IN-induced DSBs and evade cellular apoptosis (Figure 1). The virus does so very early in its infectious cycle by hijacking cellular HuR to degrade lincRNA-p21, and cripples its association with the nuclear protein binding partner hnRNP-K by spatially separating the two molecules (Figure 2). HIV-1 ensures cytoplasmic accumulation of hnRNP-K via the previously demonstrated but until now poorly understood action of MAP2K1 and ERK2 (Figure 3). Appropriate nuclear interaction between lincRNA-p21 and hnRNP-K can be restored through the inhibition of MAP2K1 or ERK2 (Figure 3), or via the combined action of Nutlin3a and enhanced expression of lincRNA-p21 (Figure 4). We propose a model (Figure 5) in which HIV-1 secures the pro-survival pathway by ensuring activated MAP2K1 continues to phosphorylate ERK2 thereby leading to cytoplasmic accumulation of hnRNP-K as well as phosphorylation of HDM2. HIV-1 had been previously reported to use this pathway to facilitate integration through the viral Env protein gp120, though the significance of these interactions was not fully appreciated until now.

Our data strongly suggest that HIV-1 can secure the pro-survival pathway in non-CD4+ T cells very early in the viral life cycle, a pathway that is unavailable to HIV-1 in CD4+ T cells due to the well-reported absence of ERK2 expression following differentiation (Fischer et al., 2005; Chang et al., 2012). In support of this, lincRNA-p21 expression was indeed increased in HIV-infected CD4+ T cells (data not shown) and Jurkat cells (Imam et al., 2015). The early stage at which HIV-1 secures that pathway is supported by the documented interaction of HIV-1 IN and Tat with ERK2 (Jacqué et al., 1998; Wolf et al., 2008; Bukong et al., 2010) as well as our own gp120 data (Figure 3), although the use of various gp120 mutants would probably illuminate this more robustly. Furthermore, our data show that HIV-1 integration does not lead to complete p53 activation, specifically phosphorylation of Ser46. As a consequence, transcription of pro-apoptotic lincRNA-p21 is decreased, and the lincRNA-p21/hnRNP-K feedback loop that suppresses pro-survival MAP2K1, is inactive.

Our data provide a glimpse of how pathogens with limited coding capacity can significantly restructure major cellular pathways using host lncRNAs. This is even more striking in the context of cell type-specific transcription: activated CD4+ T cells express low levels of MAP2K1/ERK2, and are highly sensitive to HIV-induced apoptosis. In contrast, MAP2K1/ERK2 expression remains high in macrophages (Chang et al., 2012), and these cells robustly resist viral-induced apoptosis. Thus our data suggest that the lack of ERK2 expression in CD4+ T cells may be a key reason why HIV-1 is not able to evade apoptosis in these cells, while doing so in macrophages. MAP2K1 and lincRNA-p21 are at the nexus of these pathways and their manipulation by HIV-1 adds a new layer of complexity to host-pathogen interactions.

Our findings also highlight that three discrete but tightly coordinated events are required for apoptosis to occur. Firstly, and already well-established, DNA damage such as DSBs must lead to complete activation of p53 such that downstream transcription of associated targets is initiated. Secondly, lincRNA-p21 expression levels must be enhanced. This is connected to p53 activation and HuR-mediated degradation, but can be bypassed through exogenous overexpression. Third and lastly, hnRNP-K must translocate to the nucleus to ensure its association with lincRNA-p21 and subsequent suppression of pro-survival genes such as MAP2K1. An absence of any one of these events is sufficient for the evasion of apoptosis as summarized by our findings (Figure S4F).

While it is not unusual for pathogens to manipulate host signaling pathways, our data illuminate a unique strategy whereby HIV-1 is able to control the cellular response to DNA damage via a long noncoding RNA. It has been observed that Adenovirus oncoproteins are able to inactivate the DNA repair MRN complex at viral replication centers, masking host genome instabilities that are instigated by this generally non-integrative DNA virus (Stracker et al., 2002; Shah and O'Shea, 2015). Given that viruses contribute to 20% of cancers worldwide (de Martel et al., 2012), it is important to understand how genomic instabilities are propagated following challenge, as well as how apoptosis is evaded. While HIV-1 itself does not cause cancer, our observations that HIV-mediated resistance to apoptosis occurs through manipulation of a host long noncoding RNA and its protein binding partners, reveals a novel mechanism whereby genomic integrity can be severely challenged yet cells survive. In addition, we suspect that gp120 may be initiating a general DNA damage suppression phenotype via lincRNA-p21, possibly to aid macrophage-mediated dissemination throughout the host. Lastly, given the uncontested connection between infection and cancer (de Martel et al., 2012), our data provides a novel perspective on how pathogens may increase DNA damage while simultaneously blocking apoptosis.

## Author contributions

SB and MMM designed the experiments. SB, JN, JS, MB, SP, and AKC conducted the experiments. SB and MMM analyzed the data. FB supervised SP. AS supervised MB. SB and MMM wrote the manuscript. MMM supervised the overall study.

### Conflict of interest statement

The authors declare that the research was conducted in the absence of any commercial or financial relationships that could be construed as a potential conflict of interest.

## Supplementary material

Figure S1 HIV-1 masks DNA damage, protects against additional lethal DNA damage, and prevents lincRNA-p21 upregulation. **(A)** HIV-1 integration occurs approximately 16 h post-infection of Ghost(3) reporter cells and Tat-mediated activation of an integrated LTR-driven GFP reporter can be detected approximately 48 h post-infection. **(B)** Ghost(3) cells support HIV-1 replication over time as detected by quantitative real-time RT-PCR analysis of HIV-1 Gag expression relative to the HPRT housekeeping gene and normalized to uninfected cells (mean ± SE of 3 biological replicates in triplicate). **(C)** HIV−1 infection of Ghost(3) cells induces DSBs over 48 h as detected by H2A.XpSer139 immunofluorescence staining but does not lead to caspase 3–mediated apoptosis (displayed as percentage cell survival; *n* = 3,000). Doxorubicin treatment (Doxo.) over the same time course yields extensive H2A.XpSer139 staining followed by apoptosis. **(D)** HIV–mediated DSBs require integration as addition of Raltegravir (Ralt.), an integrase inhibitor, prevents DSBs and H2A.XpSer139 staining in HIV–infected Ghost(3) cells over 48 h. **(E)** Raltegravir (Ralt.) prevents HIV−1 infection of Ghost(3) cells as detected by quantitative real–time RT–PCR analysis of HIV−1 Gag expression relative to the HPRT housekeeping gene and normalized to uninfected cells (mean ± SE of 3 biological replicates in triplicate). **(F)** Nuclear inactive p53 monomers are not phosphorylated at serine residue 46 (specific apoptotic mark) in response to HIV-1 infection of M*φ* as measured by immunofluorescence staining (p53pSer46). Nuclear activated p53 dimers are detected in Doxorubicin-treated cells. Cells were counterstained with DAPI; scale bars = 10 μM; two-tailed paired Student *T*-test, ^***^*p* < 0.001, ^**^*p* < 0.01, ^*^*p* < 0.05, NS, not significant.

Figure S2 HIV-1 manipulates lincRNA-p21's protein binding partners. **(A)** Untreated Ghost(3) cells show nuclear HuR over a 48 h time course by immunofluorescence staining. **(B)** HuR expression is significantly decreased as measured by quantitative real–time RT–PCR analysis following 48 h of exposure to siHuR in Ghost(3) cells (mean ± SE of 3 biological replicates in triplicate). **(C)** LincRNA-p21 expression increases in the absence of HuR in untreated and HIV-infected Ghost(3) cells as measured over time by quantitative real-time RT-PCR analysis relative to the HPRT housekeeping gene (mean ± SE of 3 biological replicates in triplicate). **(D)** siHuR-treated Ghost(3) cells support HIV-1 replication to the same extent as untreated cells, as indicated by GFP expression. Scale bar = 5 μM. **(E)** siHuR-treated Ghost(3) cells support HIV-1 replication as measured by quantitative real-time RT-PCR analysis of HIV-1 Gag relative to the HPRT housekeeping gene (mean ± SE of 3 biological replicates in triplicate). **(F)** Exogenous full-length lincRNA-p21 expression is significantly decreased in the presence of HIV-1 as measured over time in Ghost(3) cells by quantitative real-time RT-PCR analysis relative to HPRT housekeeping gene (mean ± SE of 3 biological replicates in triplicate). **(G)** Exogenous full-length lincRNA-p21 treated Ghost(3) cells support HIV-1 replication as measured by quantitative real-time RT-PCR analysis of HIV-1 Gag relative to the HPRT housekeeping gene (mean ± SE of 3 biological replicates in triplicate). **(H)** Exogenous full-length lincRNA-p21 expression (FL) followed by Doxorubicin treatment leads to apoptosis in Ghost(3) cells. No other treatments lead to significant apoptosis. Too few attached cells (<20) were present for statistical analysis. **(I)** Schematic representation of RNA pulldown and mass spectrometry experiments used to identify protein binding partners of lincRNA-p21 in the presence of HIV-1. Biotinylated probes targeted to lincRNA-p21 were incubated with cellular extracts, targeted using streptavidin beads, washed, resolved on a polyacrylamide gel and identified by mass spectrometry. **(J)** In uninfected Ghost(3) cells, lincRNA-p21 associated with a unique set of proteins (red circle). Similarly, in the presence of HIV-1, lincRNA-p21 associated with a different unique set of proteins (green circle). Another subset of proteins associated with lincRNA-p21 both in the presence and absence of HIV-1 but at different levels. In the presence of HIV-1, hnRNP-K (red) associated less with lincRNA-p21. In the presence of HIV-1, HuR, XRCC6 and PRKDC (green) associated more with lincRNA-p21. Cells were counterstained with DAPI; scale bars = 10 μM (**D**, 5 μM); two-tailed paired Student *T*-test, ^***^*p* < 0.001, ^**^*p* < 0.01, ^*^*p* < 0.05, NS, not significant.

Figure S3 HIV-1 requires gp120 Env and MAP2K1/ERK2 to ensure hnRNP-K's cytoplasmic localization. **(A)** Quantitative real-time RT-PCR analysis of MAP2K1 expression relative to HPRT housekeeping gene in MAP2K1 inhibitor-treated (MPK inh.), or ERK2 inhibitor-treated (ERK inh.), or Doxorubicin-treated (+Doxo.) Ghost(3) cells normalized to untreated cells (mean ± SE of 3 biological replicates in triplicate). **(B)** Quantitative real-time RT-PCR analysis of lincRNA-p21 expression relative to HPRT housekeeping gene in MAP2K1 inhibitor-treated (MPK inh.) or ERK2 inhibitor-treated (ERK inh.) Ghost(3) cells normalized to untreated cells (mean ± SE of 3 biological replicates in triplicate). **(C)** Inhibition of MAP2K1 allows for nuclear localization of hnRNP-K as measured by immunofluorescence staining, but no apoptosis occurs in treated Ghost(3) cells. **(D)** Quantification of nuclear localized hnRNP-K in infected (HIV), treated (MPK inh.) or infected and treated (HIV+MPK inh.) Ghost(3) cells shown as mean relative fluorescence intensity (RFI) ± SE of 3 biological replicates. **(E)** Inhibition of MAP2K1 allows for nuclear localization of hnRNP-K as measured by immunofluorescence staining, but no apoptosis occurs in treated M*φ*. **(F)** Quantification of nuclear localized hnRNP-K in infected (HIV), treated (MPK inh.) or infected and treated (HIV+MPK inh.) M*φ*, shown as mean relative fluorescence intensity (RFI) ± SE of 3 donors. **(G)** The percentage of apoptotic M*φ* (normalized to total uninfected population) increases significantly in cells infected with VSV-G pseudotyped HIV-1 and exposed to a MAP2K1 inhibitor (HIV VSV-G +MPK inh.). Mean ± SE of 3 donors. **(H)** Immunofluorescence staining of the Ser218/222 activation mark on MAP2K1 in M*φ* or those treated with an inhibitor (+MPK inh.) after 8 days. **(I)** HIV-1 clone BaL.01 packaged with gp120 Env (+HIV gp120) sequesters HuR in the nucleus of infected Ghost(3) cells as shown by immunofluorescence staining, while VSV-G pseudotyped HIV-1 does not. Cells transfected with a plasmid expressing only HIV-1 gp120 (+gp120) show nuclear HuR while control cells transfected with a plasmid expressing only HIV-1 Tat (+Tat) show cytoplasmic and nuclear HuR. Cells were counterstained with DAPI; scale bars = 10 μM. Two-tailed paired Student *T*-test, ^***^*p* < 0.001, ^**^*p* < 0.01, ^*^*p* < 0.05, NS, not significant.

Figure S4 Nutlin3a confirms pivotal role of hnRNP-K in apoptosis evasion by HIV-1. **(A)** Exogenous lincRNA-p21 (full-length or truncated) expression alone does not lead to nuclear hnRNP-K in the presence of HIV-1 in Ghost(3) cells. **(B)** Exogenous lincRNA-p21 (full-length or truncated) expression and Nutlin3a treatment do not lead to apoptosis in Ghost(3) cells. **(C)** Exogenous lincRNA-p21 (full-length or truncated) expression and Nutlin3a treatment do not lead to nuclear hnRNP-K in Ghost(3) cells. **(D)** The percentage of apoptotic M*φ* (normalized to total uninfected population) significantly increases in M*φ* infected with VSV-G pseudotyped HIV-1 and exposed to Nutlin3a (HIV VSV-G+Nut.3). Mean ± SE of 3 donors. **(E)** LincRNA-p21 expression is significantly increased in M*φ* infected with either gp120 or VSV-G pseudotyped HIV-1 and exposed to Nutlin3a, as detected by quantitative real-time RT-PCR analysis relative to the HPRT housekeeping gene and normalized to untreated cells (mean ± SE of 2 biological replicates in triplicate). **(F)** Summary of experimental conditions showing that only a combination of DNA damage, enhanced lincRNA-p21 expression and nuclear hnRNP-K leads to apoptosis. An absence of any one of these events, or exogenous expression of truncated lincRNA-p21 (which is unable to bind hnRNP-K), is sufficient to prevent apoptosis. Cells were counterstained with DAPI; scale bars = 10 μM. Two-tailed paired Student *T*-test, ^***^*p* < 0.001, ^**^*p* < 0.01, ^*^*p* < 0.05, NS, not significant.
