# Supplementary figures and images for: Viral Apoptosis Evasion via the MAPK Pathway by Use of a Host Long Noncoding RNA
Source: Front Cell Infect Microbiol. 2018 Aug 3;8:263. doi: 10.3389/fcimb.2018.00263 (PMC6086015; doi:10.3389/fcimb.2018.00263)

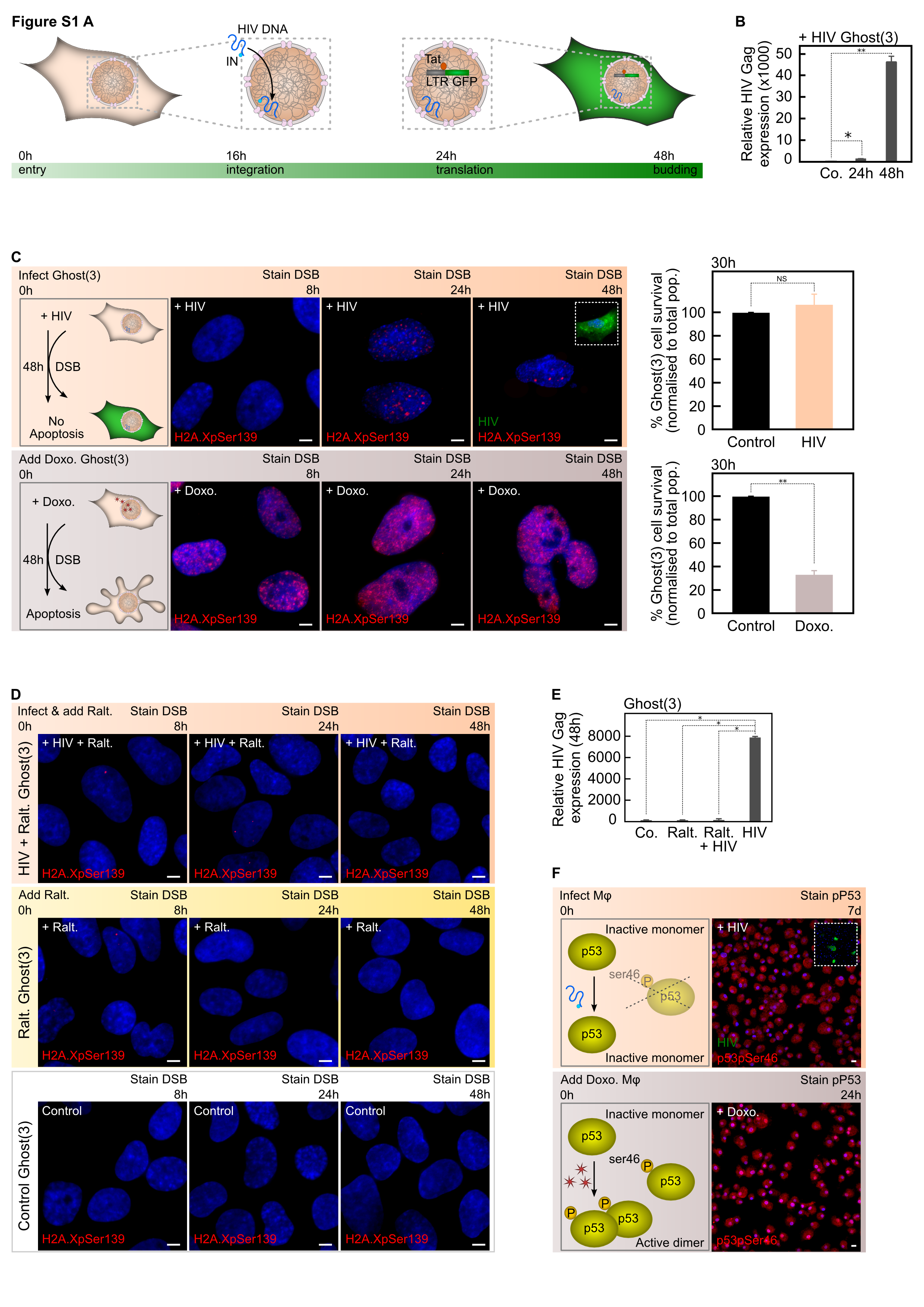

Supplement: Figure S1 — HIV-1 masks DNA damage, protects against additional lethal DNA damage, and prevents lincRNA-p21 upregulation. (A) HIV-1 integration occurs approximately 16 h post-infection of Ghost(3) reporter cells and Tat-mediated activation of an integrated LTR-driven GFP reporter can be detected approximately 48 h post-infection. (B) Ghost(3) cells support HIV-1 replication over time as detected by quantitative real-time RT-PCR analysis of HIV-1 Gag expression relative to the HPRT housekeeping gene and normalized to uninfected cells (mean ± SE of 3 biological replicates in triplicate). (C) HIV−1 infection of Ghost(3) cells induces DSBs over 48 h as detected by H2A.XpSer139 immunofluorescence staining but does not lead to caspase 3–mediated apoptosis (displayed as percentage cell survival; n = 3,000). Doxorubicin treatment (Doxo.) over the same time course yields extensive H2A.XpSer139 staining followed by apoptosis. (D) HIV–mediated DSBs require integration as addition of Raltegravir (Ralt.), an integrase inhibitor, prevents DSBs and H2A.XpSer139 staining in HIV–infected Ghost(3) cells over 48 h. (E) Raltegravir (Ralt.) prevents HIV−1 infection of Ghost(3) cells as detected by quantitative real–time RT–PCR analysis of HIV−1 Gag expression relative to the HPRT housekeeping gene and normalized to uninfected cells (mean ± SE of 3 biological replicates in triplicate). (F) Nuclear inactive p53 monomers are not phosphorylated at serine residue 46 (specific apoptotic mark) in response to HIV-1 infection of Mφ as measured by immunofluorescence staining (p53pSer46). Nuclear activated p53 dimers are detected in Doxorubicin-treated cells. Cells were counterstained with DAPI; scale bars = 10 μM; two-tailed paired Student T-test, ***p < 0.001, **p < 0.01, *p < 0.05, NS, not significant. [file Image_1.TIFF]

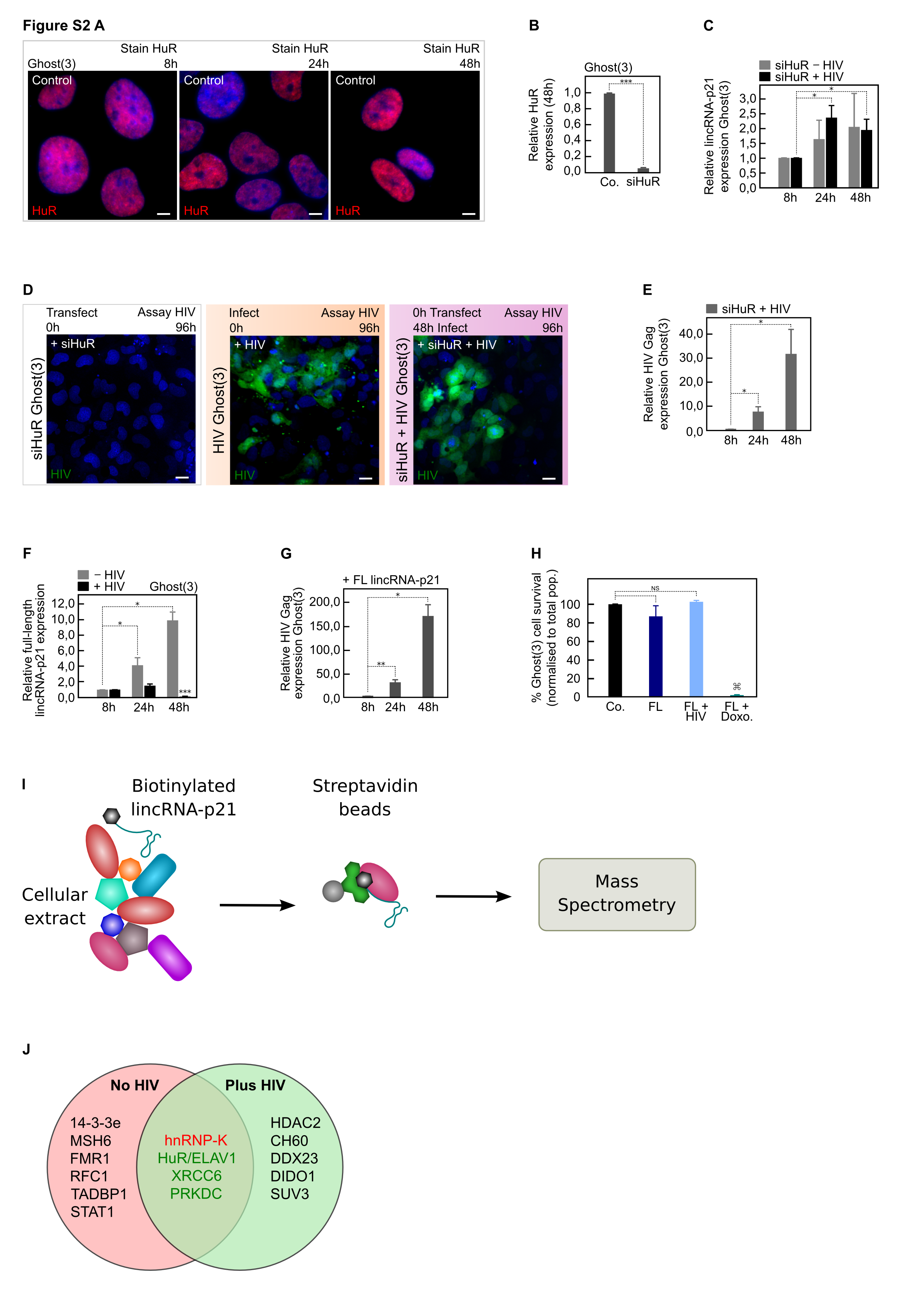

Supplement: Figure S2 — HIV-1 manipulates lincRNA-p21's protein binding partners. (A) Untreated Ghost(3) cells show nuclear HuR over a 48 h time course by immunofluorescence staining. (B) HuR expression is significantly decreased as measured by quantitative real–time RT–PCR analysis following 48 h of exposure to siHuR in Ghost(3) cells (mean ± SE of 3 biological replicates in triplicate). (C) LincRNA-p21 expression increases in the absence of HuR in untreated and HIV-infected Ghost(3) cells as measured over time by quantitative real-time RT-PCR analysis relative to the HPRT housekeeping gene (mean ± SE of 3 biological replicates in triplicate). (D) siHuR-treated Ghost(3) cells support HIV-1 replication to the same extent as untreated cells, as indicated by GFP expression. Scale bar = 5 μM. (E) siHuR-treated Ghost(3) cells support HIV-1 replication as measured by quantitative real-time RT-PCR analysis of HIV-1 Gag relative to the HPRT housekeeping gene (mean ± SE of 3 biological replicates in triplicate). (F) Exogenous full-length lincRNA-p21 expression is significantly decreased in the presence of HIV-1 as measured over time in Ghost(3) cells by quantitative real-time RT-PCR analysis relative to HPRT housekeeping gene (mean ± SE of 3 biological replicates in triplicate). (G) Exogenous full-length lincRNA-p21 treated Ghost(3) cells support HIV-1 replication as measured by quantitative real-time RT-PCR analysis of HIV-1 Gag relative to the HPRT housekeeping gene (mean ± SE of 3 biological replicates in triplicate). (H) Exogenous full-length lincRNA-p21 expression (FL) followed by Doxorubicin treatment leads to apoptosis in Ghost(3) cells. No other treatments lead to significant apoptosis. Too few attached cells (<20) were present for statistical analysis. (I) Schematic representation of RNA pulldown and mass spectrometry experiments used to identify protein binding partners of lincRNA-p21 in the presence of HIV-1. Biotinylated probes targeted to lincRNA-p21 were incubated with cellular extra [file Image_2.TIFF]

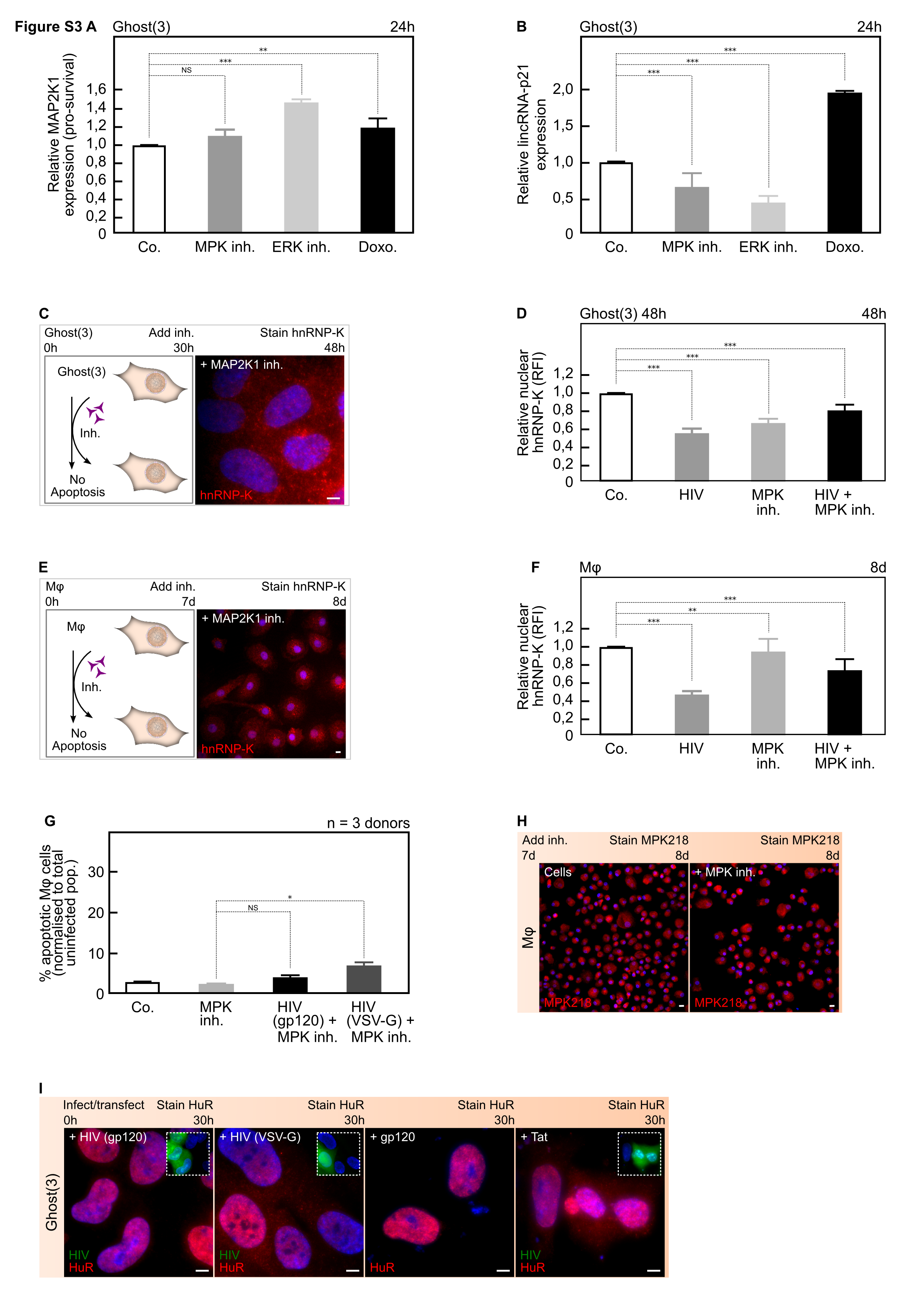

Supplement: Figure S3 — HIV-1 requires gp120 Env and MAP2K1/ERK2 to ensure hnRNP-K's cytoplasmic localization. (A) Quantitative real-time RT-PCR analysis of MAP2K1 expression relative to HPRT housekeeping gene in MAP2K1 inhibitor-treated (MPK inh.), or ERK2 inhibitor-treated (ERK inh.), or Doxorubicin-treated (+Doxo.) Ghost(3) cells normalized to untreated cells (mean ± SE of 3 biological replicates in triplicate). (B) Quantitative real-time RT-PCR analysis of lincRNA-p21 expression relative to HPRT housekeeping gene in MAP2K1 inhibitor-treated (MPK inh.) or ERK2 inhibitor-treated (ERK inh.) Ghost(3) cells normalized to untreated cells (mean ± SE of 3 biological replicates in triplicate). (C) Inhibition of MAP2K1 allows for nuclear localization of hnRNP-K as measured by immunofluorescence staining, but no apoptosis occurs in treated Ghost(3) cells. (D) Quantification of nuclear localized hnRNP-K in infected (HIV), treated (MPK inh.) or infected and treated (HIV+MPK inh.) Ghost(3) cells shown as mean relative fluorescence intensity (RFI) ± SE of 3 biological replicates. (E) Inhibition of MAP2K1 allows for nuclear localization of hnRNP-K as measured by immunofluorescence staining, but no apoptosis occurs in treated Mφ. (F) Quantification of nuclear localized hnRNP-K in infected (HIV), treated (MPK inh.) or infected and treated (HIV+MPK inh.) Mφ, shown as mean relative fluorescence intensity (RFI) ± SE of 3 donors. (G) The percentage of apoptotic Mφ (normalized to total uninfected population) increases significantly in cells infected with VSV-G pseudotyped HIV-1 and exposed to a MAP2K1 inhibitor (HIV VSV-G +MPK inh.). Mean ± SE of 3 donors. (H) Immunofluorescence staining of the Ser218/222 activation mark on MAP2K1 in Mφ or those treated with an inhibitor (+MPK inh.) after 8 days. (I) HIV-1 clone BaL.01 packaged with gp120 Env (+HIV gp120) sequesters HuR in the nucleus of infected Ghost(3) cells as shown by immunofluorescence staining, while VSV-G pseudotyped HIV-1 does not. Cells transfected [file Image_3.TIFF]

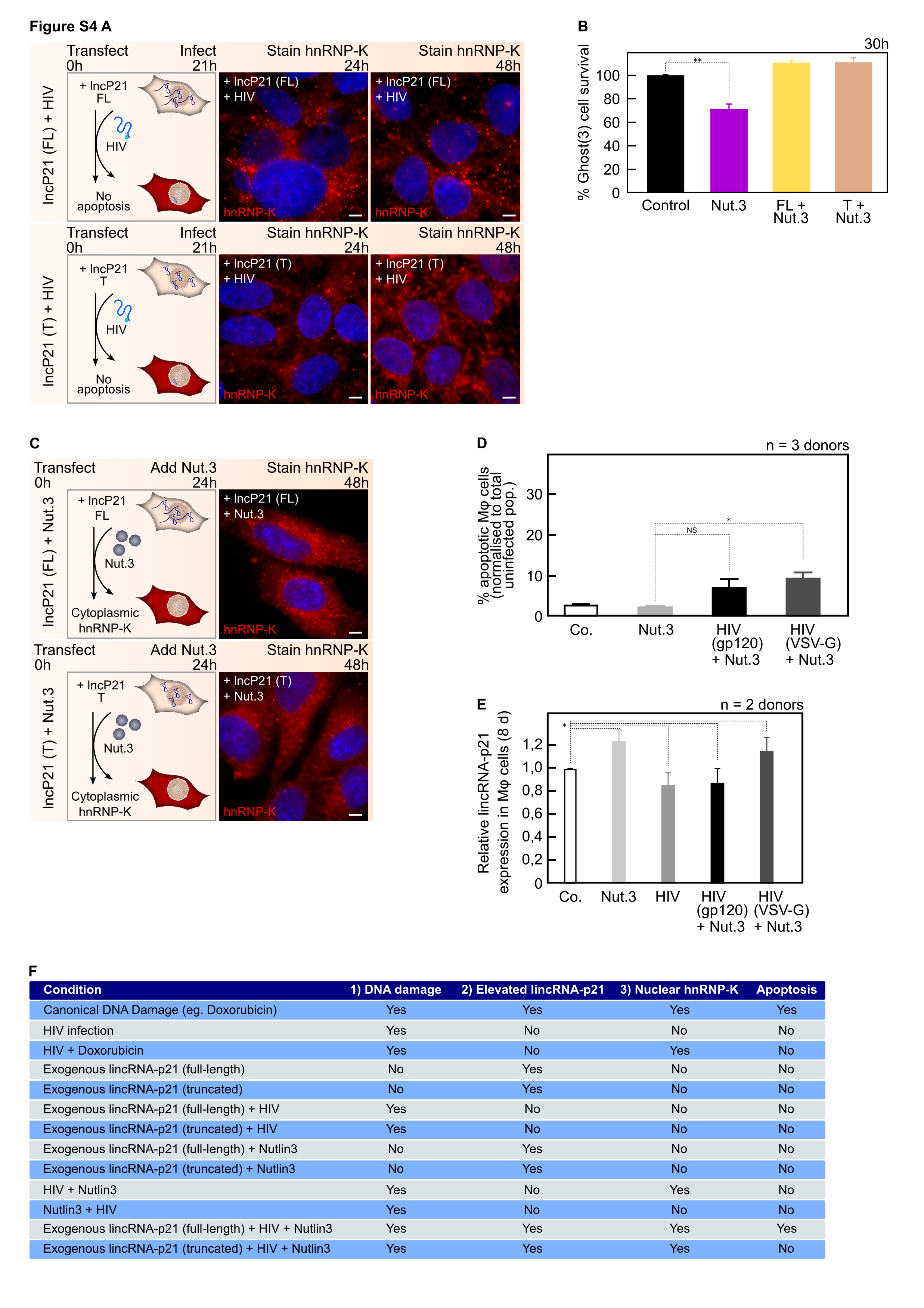

Supplement: Figure S4 — Nutlin3a confirms pivotal role of hnRNP-K in apoptosis evasion by HIV-1. (A) Exogenous lincRNA-p21 (full-length or truncated) expression alone does not lead to nuclear hnRNP-K in the presence of HIV-1 in Ghost(3) cells. (B) Exogenous lincRNA-p21 (full-length or truncated) expression and Nutlin3a treatment do not lead to apoptosis in Ghost(3) cells. (C) Exogenous lincRNA-p21 (full-length or truncated) expression and Nutlin3a treatment do not lead to nuclear hnRNP-K in Ghost(3) cells. (D) The percentage of apoptotic Mφ (normalized to total uninfected population) significantly increases in Mφ infected with VSV-G pseudotyped HIV-1 and exposed to Nutlin3a (HIV VSV-G+Nut.3). Mean ± SE of 3 donors. (E) LincRNA-p21 expression is significantly increased in Mφ infected with either gp120 or VSV-G pseudotyped HIV-1 and exposed to Nutlin3a, as detected by quantitative real-time RT-PCR analysis relative to the HPRT housekeeping gene and normalized to untreated cells (mean ± SE of 2 biological replicates in triplicate). (F) Summary of experimental conditions showing that only a combination of DNA damage, enhanced lincRNA-p21 expression and nuclear hnRNP-K leads to apoptosis. An absence of any one of these events, or exogenous expression of truncated lincRNA-p21 (which is unable to bind hnRNP-K), is sufficient to prevent apoptosis. Cells were counterstained with DAPI; scale bars = 10 μM. Two-tailed paired Student T-test, ***p < 0.001, **p < 0.01, *p < 0.05, NS, not significant. [file Image_4.TIFF]
